# Computational prediction of Pho regulons in cyanobacteria

**DOI:** 10.1186/1471-2164-8-156

**Published:** 2007-06-08

**Authors:** Zhengchang Su, Victor Olman, Ying Xu

**Affiliations:** 1 Bioinformatics Research Center and Department of Computer Science, the University of North Carolina at Charlotte, Charlotte, NC 28233, USA; 2 Computational Systems Biology Laboratory, Department of Biochemistry and Molecular Biology, University of Georgia, Athens, Georgia, USA

## Abstract

**Background:**

Phosphorus is an essential element for all life forms. However, it is limiting in most ecological environments where cyanobacteria inhabit. Elucidation of the phosphorus assimilation pathways in cyanobacteria will further our understanding of the physiology and ecology of this important group of microorganisms. However, a systematic study of the Pho regulon, the core of the phosphorus assimilation pathway in a cyanobacterium, is hitherto lacking.

**Results:**

We have predicted and analyzed the Pho regulons in 19 sequenced cyanobacterial genomes using a highly effective scanning algorithm that we have previously developed. Our results show that different cyanobacterial species/ecotypes may encode diverse sets of genes responsible for the utilization of various sources of phosphorus, ranging from inorganic phosphate, phosphodiester, to phosphonates. Unlike in *E. coli*, some cyanobacterial genes that are directly involved in phosphorus assimilation seem to not be under the regulation of the regulator SphR (orthologue of PhoB in *E coli*.) in some species/ecotypes. On the other hand, SphR binding sites are found for genes known to play important roles in other biological processes. These genes might serve as bridging points to coordinate the phosphorus assimilation and other biological processes. More interestingly, in three cyanobacterial genomes where no *sphR *gene is encoded, our results show that there is virtually no functional SphR binding site, suggesting that transcription regulators probably play an important role in retaining their binding sites.

**Conclusion:**

The Pho regulons in cyanobacteria are highly diversified to accommodate to their respective living environments. The phosphorus assimilation pathways in cyanobacteria are probably tightly coupled to a number of other important biological processes. The loss of a regulator may lead to the rapid loss of its binding sites in a genome.

## Background

Cyanobacteria are among the oldest life form on Earth. These organisms inhabit a broad range of ecological environments from fresh water, soil to diverse open oceanographic areas [1]. It is estimated that several cyanobacteria living in the open oceans contribute a significant fraction of Earth's primary production [2]. These bacteria also play important roles in the global cycling of nitrogen and phosphorus [3,4]. Therefore their activities have significant impacts on global environmental changes.

Phosphorus is one of the essential elements for all life forms, since it is required for the biosyntheses of nucleotides and phospholipids and for the functional regulation of proteins through phosphorylation. However, inorganic phosphate (P_i_), the only form of phosphorus that can be directly utilized by cells, is limiting in most ecosystems [5-7]. Thus, bacteria have evolved to develop various mechanisms to assimilate different phosphorus-containing compounds available to them. Phosphorus assimilation pathways have been relatively well studied in *E. coli *[8], and *B. subtilis *[9,10]. For instance, it is generally known that phosphorus assimilation related genes in *E. coli *are regulated by a two-component system comprising the sensor protein, histidine kinase PhoR, and the transcription factor PhoB [8]. PhoR is activated through auto-phosphorylation when the P_i _level in the environment is low. Activated PhoR then phosphorylates PhoB, thereby activating the latter. Phosphorylated PhoB can either activate or suppress the transcription of genes involved in phosphorus assimilation, resulting in changes in gene expression pattern that accommodate to the availability and the types of phosphorus-containing compounds present in the environment [8]. All the genes whose transcription is regulated by PhoB in a bacterium are collectively called a *Pho regulon*. The known members of the Pho regulon in these relatively well-studied species include genes encoding (a) the two-component system such as PhoB and PhoR in *E*. coli [8] or PhoP and PhoRin *B. subtilis *[9,10], (b) porins and transporters for transporting phosphorus-containing compounds into the cell, and (c) metabolic enzymes that are used to break down phosphorus-containing compounds to release P_i_. In *E coli*, PhoB is known to bind to at least two tandem direct repeats (Pho boxes) of 8 bp with consensus sequence CTGTACTA separated by an A/T rich 3 bp linker, located 10 bp upstream of the -10 *σ*^70 ^binding consensus TAN_3_T/A [8]. At least 8 operons comprising 39 genes in *E. coli *have been identified to be regulated by PhoB [8].

The Pho regulon in cyanobacteria has only been relatively well studied in *Synechocystis *PCC 6803 (PCC6803) [11,12]. Previous studies have shown that a two-component system comprising the sensor kinase SphS (orthologue of PhoR in *E. coli*) and the response regulator SphR (orthologue of PhoB in *E. coli*) works in a similar manner to sense the P_i _level in the environment and to regulate the expression of genes that are directly involved in phosphorus assimilation, forming a Pho regulon similar to that in *E coli *[11,12]. However, only three operons have been identified to be activated by the phosphorylated SphR, including the operons *sphX-pstS1-C1-A1-B1-B1' *and *pstS2-C2-A2-B2*, each of which encodes an ABC type high-affinity P_i _uptake system, and the operon *phoA-nucH*, where *phoA *encodes an alkaline phosphatase, and *nucA *an extracellular nuclease [12]. The singleton operon *urtA *that encodes a subunit of urea transporter is repressed under phosphorus limitation by an unknown mechanism [12]. SphR activates the transcription of genes by binding to at least three tandem direct repeats (Pho boxes) of 8 bp, but with a consensus sequence different than that in *E. coli*, namely, CTTAACCT, in the promoter regions of these genes [12]. It is unknown whether the Pho regulon in PCC6803 includes any other genes in addition to these three operons. Furthermore, although *sphR *is encoded in 15 other sequenced cyanobacterial genomes besides PCC6803, little is known about which genes in these genomes are regulated by SphR. In this work, we have predicted the Pho regulons in 16 sequenced cynaobacterial genomes that encode the *sphR *gene using an efficient phylogenetic footprinting based motif scanning algorithm [13]. Interestingly, three cyanobacterial genomes, i.e., *Prochlorococcus marinus *CCMP1375 (CCMP1375) and *Synechococcus sp. CC9311 *(CC9311) and *Synechococcus sp*. CC9902 (CC9902) that do not encode the *sphR *gene, provide us a rare and highly valuable opportunity to examine the turnover of the *cis*-regulatory binding sites after their regulators were lost during the course of evolution.

## Results

### 1. Conservation of the DNA binding domain of SphR

**Figure 1:**
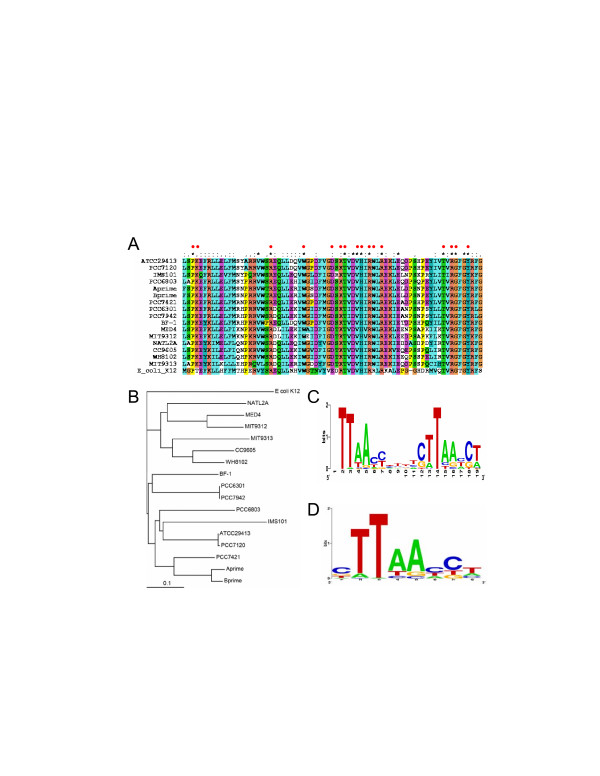
Profiles of the Pho boxes constructed by phylogenetic footprinting. A. Multiple sequence alignments of the DNA binding domains of the SphR sequences of 16 sequenced cyanobacteria and that of the PhoB of *E. coli*, generated by the ClustalX program. The red dots show the positions in PhoB sequence of *E. coli *that are in direct contact with the Pho boxes of the DNA sequence in the crystal structure of PhoB-DNA complex. B. Phylogenetic relationships of SphR of the 16 genomes in our analyses. The tree was generated using the neighbor-joining method based on the multiple sequence alignments by the ClustalX program using the default settings, and is rooted at PhoB of *E. coli *K12. Scale bar, substitutions per position. C. Logo representation of the profile of the putative two tandem repeats of Pho boxes plus the linker found by phylogenetic footprinting. D. Logo representation of the profile of the combined putative Pho boxes of the two tandem repeats shown in panel (C). Logos were generated by the Weblogo server [32].

We have identified orthologues of SphR of PCC6803 (sll0337) in 16 out of the 19 sequenced cyanobacterial genomes using the criterion described in the Methods section. Interestingly, the genomes of CCMP1375, CC9311 and CC9902 do not encode the *sphR *gene. As shown in Figure 1A, the DNA binding domain of SphR of the 16 cyanobacteria is highly conserved, suggesting that the DNA binding sites of SphRs in these species are likely to be conserved. In particular, the amino acid residues of PhoB of *E. coli *that are in direct contact with the Pho boxes (marked by red dots) as revealed by the crystal (tertiary) structure of the PhoB-DNA complex [14] are also highly conserved in the SphR sequences (Figure 1A). This may explain why both PhoB of *E. coli *and SphR of PCC6803 bind to 8 bp tandem repeats with a 3 bp linker [8,12]. We therefore infer that all the SphRs in these cyanobacterial genomes bind to *cis*-regulatory sites with a similar sequence structure. The phylogenetic relationship among these SphR sequences is shown in Figure 1B.

### 2. Profiles of the SphR binding sites predicted by phylogenetic footprinting

When the phylogenetic footprinting technique is applied to the pooled 50 upstream regions (data not shown) of the operons whose genes are orthologous to those that are regulated by SphR in PCC6803, highly conserved two tandem repeats with a linker are identified in 30 these sequences by our motif finding program CUBIC [15] with a cutoff score at *p *< 0.001. We assume that these motifs are putative Pho boxes, and are shown in Table 1. The three known SphR binding sites in PCC6803 are accurately recovered (Table 1), suggesting that most of the identified motifs are likely to be true Pho boxes. Shown in Figure 1C is the logo representation of the sequence profile of these putative two tandem Pho boxes plus the linker; Figure 1D is the logo representation of the sequence profile of the combined putative Pho boxes of the two tandem repeats shown in 1C. However, such conserved two tandem repeats are not found for any orthologous genes from CC9605 MIT9313, CC9311 and CC9902, suggesting that these genes do not bear Pho boxes or their Pho boxes differ largely from the conserved ones, which cannot be identified by CUBIC. Interestingly, CUBIC finds high scoring tandem Pho boxes for the *pstC-A-B *operon in CCMP1375, although this genome does not encode the *sphR *gene, thus they are unlikely to be functional.

**Table 1:** Genes with two tandem Pho boxes found in their promoter regions by phylogenetic footprinting

Genome	Rank^1^	Tanscription unit	Names	Pho Boxes^2^	Positon^3^	Score^4^
ATCC29413	7	*Ava_2541*	*phoA*	**GTTAATCTtcaGTTAACCT**aaaTTTATATT	-111	9.07
	11	*Ava_4516 Ava_4515 Ava_4514 Ava_4513*	*pstS2 pstC2 pstA2 pstB2*	TATTTGTAtat**TTTAACTTgaaATTAACCA**	-403	8.75
		*Ava_4512*	*-*			
	17	*Ava_2477*	*pstS1 pstC1 pstA1, pstB1'*	TTTTTCCTtgt**TTTAACCTttcGTTAACCT**	-66	8.54
	27	*Ava_3716*	*sphX*	**CTTTACTGataCATCAACT**tttTTTGATGA	-104	8.29
	47	*Ava_4728*	*nucH*	TTTTACCTttt**CCTATTATtctCTTAATCT**	-174	8.04
Aprime	3	*CYA_1552 CYA_1553 CYA_1554 CYA_1555*	*pstS pstC pstA pstB*	AATAACCTgaa**TTTAACCTcttGTTAACCA**	-175	10.11
		*CYA_1556 CYA_1557*	--			
Bprime	10	*CYB_2765 CYB_2766*	*nucH -*	**CTTAAGCGtacCTTAACTG**cccCTTTACTC	-91	9.49
BF-1	2	*tlr2164 tlr2165 tlr2166 tlr2167*	*sphX pstC pstA pstB*	TTTAAACAaa**cTTTTACCTtctCTTAACTT**	-23	9.91
IMS101	21	*Tery_3534*	*pstS*	TTTGATATttt**TTTAACCTgttCTTAATCT**	-164	8.18
PCC6301	1	*syc0545_d*	*nucH*	**TTTAAAGTgctGTTAATCC**ttcCTTTACCG	-276	9.76
	2	*syc1661_d syc1662_d syc1663_d syc1664_d*	*sphX pstS pstC pstA*	**CTTAGGGTcgcCTTAATAG**gctGTTAAACT	-48	9.75
		*syc1665_d syc1666_d*	*pstB pnp*			
	4	*syc0163_d*	*phoA*	TTTAACTAttt**CATAATCTattCTCAATCT**	-216	9.41
PCC6803	1	*sll0679*	*sphX*	*TTTAACCAaac* ***CTTTACTAgggCTTAACCT***	-105	10.31
	2	*slr1247 slr1248 slr1249 slr1250*	*pstS2 pstC2 pstA2 pstB2*	***CTTAATTCtatCTTAATTTc *** *gaCTTAATCA*	-303	10.10
	7	*sll0654 sll0656*	*phoA nucH*	***CTTAACCTtttCATAGTCT *** *aacCATAAGTT*	-156	8.80
	14	*sll0680 sll0681 sll0682 sll0683 sll0684*	*pstS1 pstC1 pstA1 pstB1 pstB1'*	ATTCCATAgac**CTTAACCTtccCTTTACCA**	-73	8.46
PCC7120	6	*alr5291*	*phoA*	**ATTAATCTttaGTTAACCT**gaaTTTATATT	-223	9.25
	10	*all4575 all4574 all4573 all4572*	*pstS1 pstC1 pstA1 pstB1*	TTTTCTCTtga**TTTAACCTttcGTTAACCT**	-64	8.97
	38	*alr1094 alr1095*	*sphX gap3*	**CTTTACTAataCATCAACT**tttATTGATGA	-106	8.19
	51	*alr0276*	*nucH*	**TTTTACCTtctCCTATTAT**tctCTTAATCT	-178	8.05
PCC7421	31	*glr0445 glr0446 glr0447 glr0448*	*pstS pstC pstA pstB*	**GTTAACCTtgcCTTAATC**GaatTTCGTTAC	-80	8.64
PCC7942	1	*Synpcc7942_1000*	*nucH*	TTTAAAGTgct**GTTAATCCttcCTTTACCG**	-310	9.72
	2	*Synpcc7942_2445*	*sphX*	**CTTAGGGTcgcCTTAATAG**gctGTTAAACT	-46	9.71
	4	*Synpcc7942_1392*	*phoA*	TTTAACTAttt**CATAATCTattCTCAATCT**	-214	9.37
		*Synpcc7942_2441*	*-*			
CCMP1375	18	*Pro0598 Pro0597 Pro0596*	*pstC pstA pstB*	**CTTAACTCtctCTTAAACA**tgcACTTGATA	-47	7.36
MED4	14	*PMM0710*	*pstS*	TTTAACTAgcc**CTTAATCAtttCTTATATT**	-245	8.07
	24	*PMM0723 PMM0724 PMM0725*	*pstC pstA pstB*	TTTATATAtac**CTTTAACCcttCTTAAAGT**	-48	7.67
MIT9312	8	*PMT9312_0722*	*pstS*	TTTAAAGAgaa**CTTAAACTtccCTTAAATT**	-231	8.49
NATL2A	13	*PMN2A_0311 PMN2A_0310 PMN2A_0309*	*pstC pstA pstB*	ATTAACCCtct**CTTAGTGAaaaATTTGAAA**	-47	7.52
		*PMN2A_0308 PMN2A_0307*	--			
WH8102	1	*SYNW2391 SYNW2390*	*- phoA*	TTTGATCAgat**CTTAAACTattCCTAACTT**	-59	11.18

1. The rank of three tandem Pho boxes recovered by our scanning algorithm.

2. Bold, the two tandem Pho boxes identified by phylogenetic footprinting; light, the additional Pho box found by our scanning algorithm; Italics, known Pho boxes.

3. Positions of the Pho boxes relative to the first codon in the operon.

4. The scores of the three tandem Pho boxes recovered by our scanning algorithm.

### 3. Genome wide prediction of SphR binding sites in each genome

**Figure 2:**
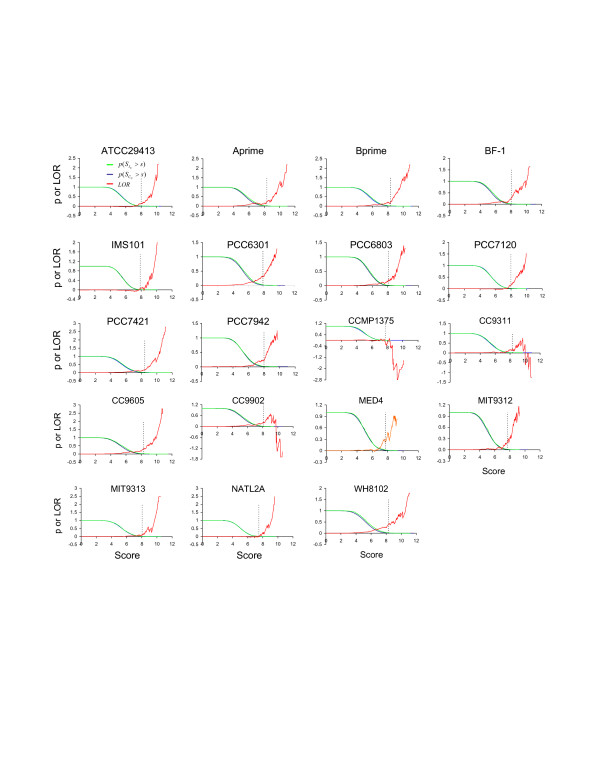
Genome wide prediction of Pho regulons in the 19 sequenced cyanobacterial genomes. Green lines represent the probability (*p*(SIU
 MathType@MTEF@5@5@+=feaafiart1ev1aaatCvAUfKttLearuWrP9MDH5MBPbIqV92AaeXatLxBI9gBaebbnrfifHhDYfgasaacH8akY=wiFfYdH8Gipec8Eeeu0xXdbba9frFj0=OqFfea0dXdd9vqai=hGuQ8kuc9pgc9s8qqaq=dirpe0xb9q8qiLsFr0=vr0=vr0dc8meaabaqaciaacaGaaeqabaqabeGadaaakeaacqWGtbWudaWgaaWcbaGaemysaK0aaSbaaWqaaiabdwfavbqabaaaleqaaaaa@308D@ > *s*)) that putative three tandem Pho boxes found in the promoter region IU(g1,...,gn)
 MathType@MTEF@5@5@+=feaafiart1ev1aaatCvAUfKttLearuWrP9MDH5MBPbIqV92AaeXatLxBI9gBaebbnrfifHhDYfgasaacH8akY=wiFfYdH8Gipec8Eeeu0xXdbba9frFj0=OqFfea0dXdd9vqai=hGuQ8kuc9pgc9s8qqaq=dirpe0xb9q8qiLsFr0=vr0=vr0dc8meaabaqaciaacaGaaeqabaqabeGadaaakeaacqWGjbqsdaWgaaWcbaGaemyvauLaeiikaGIaem4zaC2aaSbaaWqaaiabigdaXaqabaWccqGGSaalcqGGUaGlcqGGUaGlcqGGUaGlcqGGSaalcqWGNbWzdaWgaaadbaGaemOBa4gabeaaliabcMcaPaqabaaaaa@3AB7@ of an operon *U*(*g*_1_,..., *g*_*n*_) have a score greater than *s *(*s *is a positive number); blue lines represent the probability (*p*(SCU
 MathType@MTEF@5@5@+=feaafiart1ev1aaatCvAUfKttLearuWrP9MDH5MBPbIqV92AaeXatLxBI9gBaebbnrfifHhDYfgasaacH8akY=wiFfYdH8Gipec8Eeeu0xXdbba9frFj0=OqFfea0dXdd9vqai=hGuQ8kuc9pgc9s8qqaq=dirpe0xb9q8qiLsFr0=vr0=vr0dc8meaabaqaciaacaGaaeqabaqabeGadaaakeaacqWGtbWudaWgaaWcbaGaem4qam0aaSbaaWqaaiabdwfavbqabaaaleqaaaaa@3081@ > *s*)) that putative three tandem Pho boxes found in a randomly chosen coding region CU(g1,...,gn)
 MathType@MTEF@5@5@+=feaafiart1ev1aaatCvAUfKttLearuWrP9MDH5MBPbIqV92AaeXatLxBI9gBaebbnrfifHhDYfgasaacH8akY=wiFfYdH8Gipec8Eeeu0xXdbba9frFj0=OqFfea0dXdd9vqai=hGuQ8kuc9pgc9s8qqaq=dirpe0xb9q8qiLsFr0=vr0=vr0dc8meaabaqaciaacaGaaeqabaqabeGadaaakeaacqWGdbWqdaWgaaWcbaGaemyvauLaeiikaGIaem4zaC2aaSbaaWqaaiabigdaXaqabaWccqGGSaalcqGGUaGlcqGGUaGlcqGGUaGlcqGGSaalcqWGNbWzdaWgaaadbaGaemOBa4gabeaaliabcMcaPaqabaaaaa@3AAB@ with the same length as IU(g1,...,gn)
 MathType@MTEF@5@5@+=feaafiart1ev1aaatCvAUfKttLearuWrP9MDH5MBPbIqV92AaeXatLxBI9gBaebbnrfifHhDYfgasaacH8akY=wiFfYdH8Gipec8Eeeu0xXdbba9frFj0=OqFfea0dXdd9vqai=hGuQ8kuc9pgc9s8qqaq=dirpe0xb9q8qiLsFr0=vr0=vr0dc8meaabaqaciaacaGaaeqabaqabeGadaaakeaacqWGjbqsdaWgaaWcbaGaemyvauLaeiikaGIaem4zaC2aaSbaaWqaaiabigdaXaqabaWccqGGSaalcqGGUaGlcqGGUaGlcqGGUaGlcqGGSaalcqWGNbWzdaWgaaadbaGaemOBa4gabeaaliabcMcaPaqabaaaaa@3AB7@ have a score greater than *s *(*s *is a positive number); Red lines represent the log-odds ratio function defined as *LOR*(*s*) = ln(*p*(SIU
 MathType@MTEF@5@5@+=feaafiart1ev1aaatCvAUfKttLearuWrP9MDH5MBPbIqV92AaeXatLxBI9gBaebbnrfifHhDYfgasaacH8akY=wiFfYdH8Gipec8Eeeu0xXdbba9frFj0=OqFfea0dXdd9vqai=hGuQ8kuc9pgc9s8qqaq=dirpe0xb9q8qiLsFr0=vr0=vr0dc8meaabaqaciaacaGaaeqabaqabeGadaaakeaacqWGtbWudaWgaaWcbaGaemysaK0aaSbaaWqaaiabdwfavbqabaaaleqaaaaa@308D@ > *s*)/*p*(SCU
 MathType@MTEF@5@5@+=feaafiart1ev1aaatCvAUfKttLearuWrP9MDH5MBPbIqV92AaeXatLxBI9gBaebbnrfifHhDYfgasaacH8akY=wiFfYdH8Gipec8Eeeu0xXdbba9frFj0=OqFfea0dXdd9vqai=hGuQ8kuc9pgc9s8qqaq=dirpe0xb9q8qiLsFr0=vr0=vr0dc8meaabaqaciaacaGaaeqabaqabeGadaaakeaacqWGtbWudaWgaaWcbaGaem4qam0aaSbaaWqaaiabdwfavbqabaaaleqaaaaa@3081@ > *s*)). The doted vertical line in each panel shows the value of *s*, such that *p*(SCU
 MathType@MTEF@5@5@+=feaafiart1ev1aaatCvAUfKttLearuWrP9MDH5MBPbIqV92AaeXatLxBI9gBaebbnrfifHhDYfgasaacH8akY=wiFfYdH8Gipec8Eeeu0xXdbba9frFj0=OqFfea0dXdd9vqai=hGuQ8kuc9pgc9s8qqaq=dirpe0xb9q8qiLsFr0=vr0=vr0dc8meaabaqaciaacaGaaeqabaqabeGadaaakeaacqWGtbWudaWgaaWcbaGaem4qam0aaSbaaWqaaiabdwfavbqabaaaleqaaaaa@3081@ > *s*) <*0.01*, and *s *is the cutoff for SphR binding site predictions. *p*(SCU
 MathType@MTEF@5@5@+=feaafiart1ev1aaatCvAUfKttLearuWrP9MDH5MBPbIqV92AaeXatLxBI9gBaebbnrfifHhDYfgasaacH8akY=wiFfYdH8Gipec8Eeeu0xXdbba9frFj0=OqFfea0dXdd9vqai=hGuQ8kuc9pgc9s8qqaq=dirpe0xb9q8qiLsFr0=vr0=vr0dc8meaabaqaciaacaGaaeqabaqabeGadaaakeaacqWGtbWudaWgaaWcbaGaem4qam0aaSbaaWqaaiabdwfavbqabaaaleqaaaaa@3081@ > *s*) is also used to estimate the *p *value.

We have predicted all possible SphR binding sites in each of the 19 sequenced genomes by scanning their genome sequences with the two profiles of the Pho boxes that we have constructed above (shown in Figure 1C and 1D), assuming that a SphR binding site consists of three Pho boxes with two 3 pb linkers. Given profiles of multiple co-occurring binding sites, our binding-site scanning algorithm computes a log-odds ratio function *LOR(s) *of the scores (*s*) of the candidate sites in a genome. LOR is the ratio of the probability of finding a SphR binding site in the intergenic regions divided by the probability of finding a SphR binding sites in randomly chosen coding regions. We use coding regions as control sequence because it is less likely that functional SphR binding sites would occur in coding regions (for details see Methods). We have argued that our scoring function captures the essence of true *cis*-binding sites in a genome so that if the genome contains functional binding sites described by the profiles, its *LOR(s) *will become positive when the score value *s *increases beyond some threshold; and the larger a *LOR(s) *value is, the higher the confidence for the prediction is [13]. In contrast, if a genome only contains similar sequences occurring by chance, then its *LOR(s) *will be around 0 or lower independent of the increase of the score value *s *[13]. As shown in Figure 2, when *s *increases beyond a certain value, the *LOR(s) *becomes positive for the 16 genomes that encode the *sphR *gene, and the *LOR(s) *values are generally high, suggesting that these 16 genomes contain more high scoring SphR binding sites than one would expect by chance, thus these high scoring sites are likely to be functional. In contrast, the *LOR(s) *for the genomes of CCMP1375, CC9311 and CC9902, which do not encode the *sphR *gene, becomes negative when *s *increases beyond a certain value, indicating that the putative SphR binding sites found in the intergenic regions of these genomes are likely to occur by chance. Therefore they are unlikely to be functional. We argue that the lack of strong signals for possible SphR binding sites in CCMP1375, CC9311 and CC9902 is unlikely caused the lack of representative Pho boxes from these genomes in the profiles used in our scanning algorithm. Our profiles do not contain any representative sites from CC9605 and MIT9313 (Table 1), yet their *LOR(s)*' are still positive. In contrast, though CCMP1375 has representative sites in our profiles (Table 1), its *LOR(s) *is negative. We conclude that our algorithm is not biased towards the genomes from which the profiles are constructed and it is robust enough to uncover true SphR binding sites in closely related cyanobacteria even when no representative site from them is included in the profiles. In addition, the results show that our scanning algorithm can tolerate some level of noise caused by including non-functional sites in the profiles. Hence, we conclude that CCMP1375, CC9311 and CC9902 are unlikely to contain functional SphR binding sites represented by the profiles. On the other hand, we have predicted putative members of the Pho regulons in the other 16 genomes based on the presence of a putative SphR binding site found in their promoter regions at a statistical significance level of *p *< 0.01 (see Methods). These results are listed in the Additional file 1: Table 1s-16s. The most interesting observations from these predictions are summarized below.

### 4. Pho regulon members that are directly involved in phosphorus assimilation process

#### 4.1 Two-component system sphS/sphR genes

**Figure 3:**
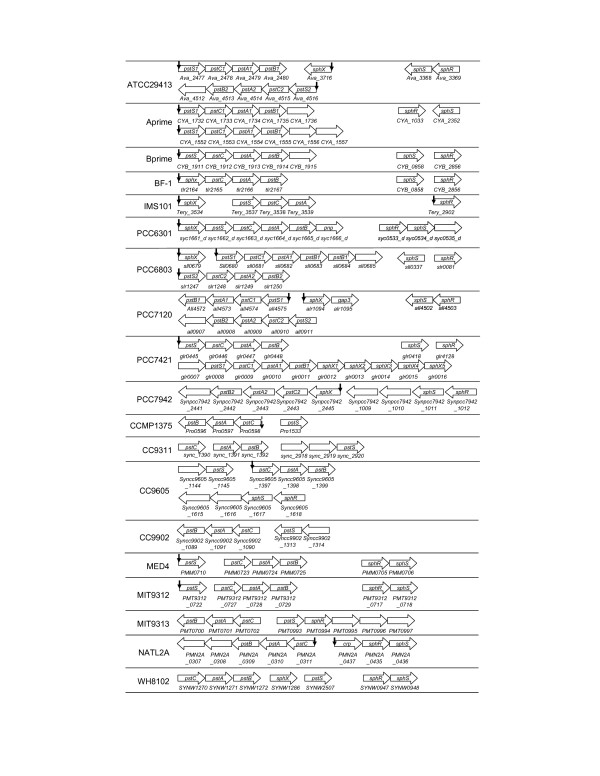
Genomic organization of the two-component system *sphS/sphR *genes and the P_i _uptake system *pst *genes in cyanobacteria. A vertical arrow indicates that three tandem Pho boxes are found for the predicted operon with *p *< 0.01. A doted arrow represents a non-functional SphR binding site. The order of the operons does not reflect their actual positions on the chromosome, but rather it is a schematic illustration of the operons.

The phosphorus sensor kinase *sphS *and response regulator *sphR *genes are predicted to be in the same operon in ATCC29413, PCC6301, PCC7120, PCC7942, CC9605, MED4, MIT9312, NATL2A and WH8102, but they are clearly split into separate operons in Aprime, Bprime, BF-1, PCC6803 and PCC7421 (Figure 3). In addition, the *sphS *gene is not encoded in IMS101; and the gene in MIT9313 is frame-shifted [16,17], and therefore is unlikely to be functional. Thus, SphS in IMS101 and MIT9313 may be activated by other regulators as suggested for MIT9313 where multiple regulatory genes are induced by P starvation [17]. High scoring tandem Pho boxes are found for the singleton operon *sphR *in IMS101 (*Tery_2902*, Table 5s and Figure 3) and for the operon *crp-sphR-sphS *in NATL2A (*PMN2A_0435-0437*, Table 15s and Figure 3), suggesting that these operons are regulated by SphR in their respective genomes. However, the *sphs/sphR *genes in the other cyanobacterial genomes are unlikely to be regulated by SphR. Thus the auto-regulation of the *phoBR *operon found in *E. coli *is not a general rule.

#### 4.2. High affinity ABC-type P_i _pst transporter complex genes

All the 19 genomes analyzed in this study encode at least one Pst transporter complex. In most of these genomes, the complex is composed of the phosphate binding subunit PstS, the transmembrane subunits PstC and PstA, and the ATP-binding subunit PstB, whose genes form an operon in the form of *pstS-C-A-B*. However, the *pstS *gene is split out to form a singleton operon in CCMP1375, CC9905, MED4, MIT9312, and MIT9313 (Figure 3). Furthermore, in the genomes of ATCC29413, IMS101, PCC6301, PCC6803, PCC7421, PCC7120, PCC7942, BF-1 and WH8102, the Pst transporter complex includes an additional phosphate binding subunit SphX. The *sphX *gene in BF-1, PCC6301 and PCC7942 forms an operon with the genes of the other subunits of the complex in the form of *sphX-pstS-C-A-B *(Figure 3). Interestingly, multiple copies of the sphX gene are found in downstream of one of the two *pst *operons in PCC7421 (Figure 3). High scoring tandem Pho boxes are found for most operons that encode one or more *pst *genes (Figure 3), except for those in the genomes CC9902, CC9311, MIT9313 and WH8102, where no Pho box is found for the operon *pstC-A-B *and the *pstS *gene. As mentioned before, Pho boxes are found for the *pstC-A-B *operon in CCMP1375 through phylogenetic footprinting, but they are unlikely to be functional due to the lack of *sphR *gene in the genome. The reason why these Pho boxes remain after *sphR *was lost is unknown. On the other hand, while we did not find any tandem Pho boxes for the *pstC-A-B *operon in CC9905 using phylogenetic footprinting (Table 1), our scanning algorithm identifies a putative SphR binding site in its promoter region with a score value only marginally above the cutoff (Table 11s). The absence of SphR binding sites for the *pstS *gene and the *pstC-A-B *operon in CC9311 and CC9902 might be due to the missing of the *sphR *gene in their genomes as discussed below. The lack of SphR binding sites for the *pstC-A-B *operon and the *pstS *gene in MIT9313 and WH8102 might be due to the relative abundance of phosphate in their environments as evidenced by the loss of the *sphS *gene in the MIT9313 genome. In addition, while experiments have shown that the gene cluster *sphX-pstS1-C1-A1-B1-B1' *in PCC6803 form an operon that is regulated by SphR through the three tandem Pho boxes in the upstream of the *sphX *gene [12], we predicted that *sphX *and *pstS1-C1-A1-B1-B1' *can form sub-transcriptional units, and a high scoring SphR binding site is found in the upstream intergenic region of the *pstS1 *gene. The possible function of this putative SphR binding site warrants further experimental investigation.

#### 4.3. Phosphonate transporter complex and C-P lyase genes

**Figure 4:**
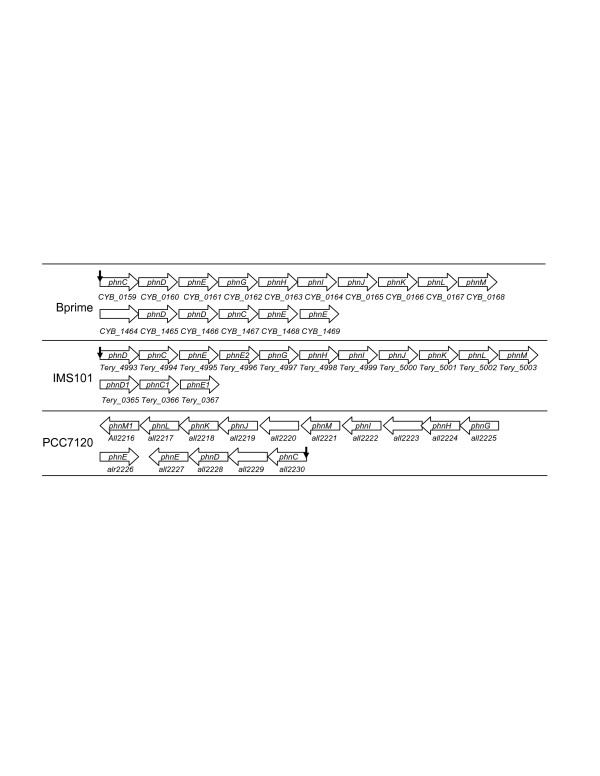
Genomic organization of the *phn *genes in cyanobacteria. A vertical arrow indicates that three tandem Pho boxes are found for the predicted operon with *p *< 0.01. The order of the operons does not reflect their actual positions on the chromosome, but rather it is a schematic illustration of the operons.

Orthologous genes of the phosphonate transporter complex and the C-P lyase in *E. coli *are found in the genomes of PCC7120, IMS101 and Bprime, suggesting that these species are capable of utilizing phosphonates as a phosphorus source for growth. The importance of phosphonates for the prevalence of IMS101 in the vast open oceans has recently been experimentally demonstrated [4]. Although in *E. coli*, genes encoding the phosphonate transporter system (*phnCDE*) and those encoding the C-P lyase complex (*phnFGHIJKLMNOP*) are located in the same operon in the form of *phnC-D-E-F-G-H-I-J-K-L-M-N-O-P *and are regulated by PhoB [18,19], the *phn *genes are split into two operons in Bprime and PCC7120, and the *phnCDE *genes are duplicated in IMS102 (Figure 4). High scoring SphR binding sites are found for the predicted operons *phnC-D-E-G-H-I-J-K-L-M *in Bprime, *phnD-C-E-E2-G-H-I-J-K-L-M *in IMS101 and *phnC-D-all229-E *in PCC7120, suggesting that they are likely to be regulated by SphR. These results are consistent with the findings in IMS101 that when tested, the *phnD *and *phnJ *genes were activated by a phosphorus-deficient medium [4] in which SphR was presumably activated by an unknown regulator.

#### 4.4. Phosphatase genes

**Figure 5:**
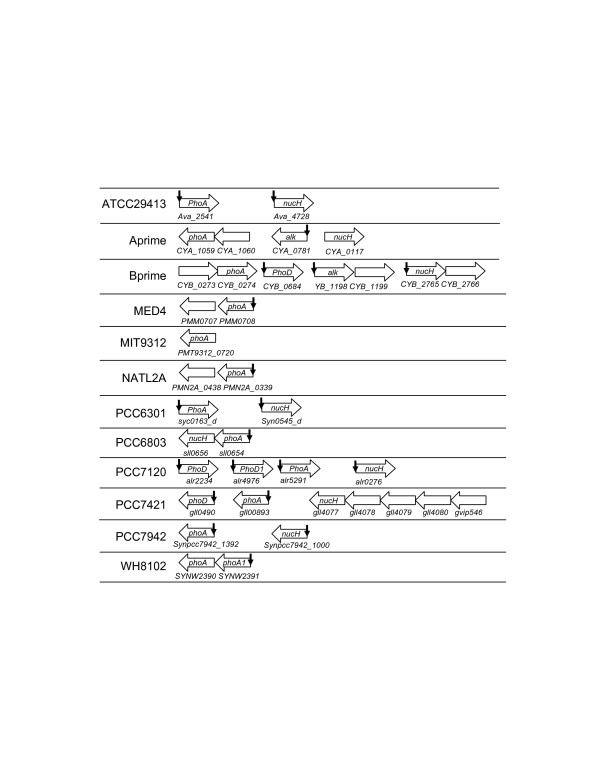
Genomic organization of alkaline phophosphatases and extracellular nuclease *nucH *genes in cyanobacteria. A vertical arrow indicates that three tandem Pho boxes are found for the predicted operon with *p *< 0.01. The order of the operons does not reflect their actual positions on the chromosome, but rather it is a schematic illustration of the operons. The label *alk *represents an alkaline phosphatase of an uncharacterized type.

Most of the 19 cyanobacterial genomes encode alkaline phosphatase genes, possibly of different families. The PhoA family members are found in ATCC29413 (*Ava_2541*), Aprime (*CYA_1059*), Bprime (*CYB_0274*), MED4 (*PMM0708*), MIT9312 (*PMT9312_0720*), NATL2A (*PMN2A_0439*), PCC6301 (*syc0163_d*), PCC6803 (*sll0654*), PCC7120 (*alr5291*), PCC7421 (*gll0893*), PCC7942 (*Syncc7942_1392*), and WH8102 (*SYNW2390 *and *SYNW2391*), mostly as a singleton operon except for Aprime, Bprime, MED4, NATL2A, PCC6803 and WH8102, in which the *phoA *gene is predicted to form an operon with other genes (Figure 5). High scoring SphR binding sites are found in their promoter regions except for *CYA_1059 *in Aprime, *CYB_0274 *in Bprime and *PMT9312_0720 *in MIT9312 (Figure 5). Furthermore, high scoring SphR binding sites are found for the PhoD family members encoded in PCC7120 (*alr4976 *and *alr2234*) and PCC7421 (*gll0490*), and for other families of alkaline phosphatases encoded in Bprime (*CYB_1198*) and Aprime (*CYA_0781*) (Table 5). These results suggest that as in *E. coli*, alkaline phosphatases in cyanobacteria play an important role in utilizing the P_i _moiety in various organic compounds, and this process is probably highly regulated by SphR in most species/ecotypes.

#### 4.5. Extracellular nuclease nucH gene

It has been shown that the *phoA-nucH *operon in PCC6803 is activated under P limitation, presumably through binding of phosphorylated SphR to the four tandem Pho boxes in its promoter region [12], suggesting that NucH might play a crucial role in the utilization of the P_i _moiety in the nucleic acids in its environment. However, the *nucH *gene is not found in the genomes of BF-1, CCMP1375, MED4, MIT9312, MIT9313, NATL2A CC9602, CC9605, WH8102, which might reflect the fact that the presence of nucleic acids in their ecological niche is less likely, such as in the oligotrophic open oceans. In the genomes where the *nucH *gene (Figure 5) is encoded, it is predicted to form a singleton operon except for PCC6803, PCC7421 and Bprime. High scoring SphR binding sites are found for the *nucH *genes in the genomes of ATTCC29413 (*Ava_2698*, Table 1s, and Figure 5), PCC6301 (*syc0545_d*, Table 6s), PCC7120 (*alr0276*, Table 8s) and PCC7942 (*Synpcc7942_1000*, Table 10s, Figure 5), suggesting that they are under the regulation of SphR in these species/ecotypes.

### 5. Putative Pho regulon members likely to be involved in phosphorus assimilation related global responses

It is interesting that high scoring SphR binding sites are found for numerous genes that might not be directly involved in the phosphorus assimilation pathway but rather in some other important biological processes, such as photosynthesis, carbon fixation and nitrogen assimilation (Table 2). We have also predicted that three other classes of genes bear SphR binding sites, namely, transporters/porins, kinases and transcription factors (Table 2). In addition, high scoring SphR binding sites are found for numerous conserved hypothetical genes across all the cyanobacterial genomes that encode the sphR gene, which are listed in Table 1s-16s. Their specific functions deserve further experimental characterization.

**Table 2:** Genes bearing a putative SphR binding site with unknown function in P assimilation

Genomes	Photosynthesis	Carbon fixation	Nitrogen assimilation	Transporters or porins	Kinases	Transcription factors
ATCC29413					*Ava_0064* *Ava_2524*	*Ava_2324*
Aprime	*CYA_0229*	*CYA_2357*				*CYA1541* *CYA_0061*
Bprime				*CYB_1898* *CYB_2516*	*CYB_2465*	
BF-1						*tll1021* *tll1024* *tll1025*
IMS101				*Tery_2377* *Ter_4515*	*Tery4221*	*Tery_2828*
PCC6301	*syc_2044_d-2045_d* *syc_1093_c*		*syn_1338_c*			*syc1747_d* *syc0684_c* *syc0685_c*
PCC6803	*slr1181*		*slr0753*			*Slr1181* *sll1555*
PCC7120	*alr0528-0537*		*glr3061*	*all3132*	*all0542* *alr1171* *alr5272*	*alr1170* *alr5224* *all0638* *all0637*
PCC7421	*gvip499*	*gll3548*		*gll0367*		
PCC7942	*Synpcc7942_0424* *Synpcc7942_2048*		*Synpcc7942_0169*		*Synpcc7942_1356-1357*	*Synpcc7942_2356* *Synpcc7942_1355*
	*-2049*					
CC9605	*Syncc9605_1756* *Syncc9605_1831* *Syncc9605_0307* *Syncc9605_0442-0444* *Syncc9605_2579*					
MED4		*PMM1438-1439*			*PMM0709*	*PMM0572*
MIT9312		*PMT9312_1542-1550*			*PMT9312_0721*	*PMT9312_0577*
MIT9313	*PMT1046*					
NATL2A						*PMN2A_0435*
WH8102	*SYNW0817* *SYNW1999-22001*				*SYNW0246*	*SYNW1019*

## Discussion

### 1. Degradation of SphR *cis*-binding sites after SphR was lost

The low *LOR(s) *values of the putative SphR binding sites in the genomes of CCMP1375, CC9311 and CC9902, in which no *sphR *gene is found, indicate that these genomes might not contain functional SphR binding sites. Since all the CCMP1375, CC9311 and CC9902 genomes encode other phosphorus assimilation related genes, such as the *pst *transporter genes, which are in general regulated by SphR in the other 16 cyanobacterial genomes that encode the *sphR *gene, it is reasonable to assume that the last common ancestor of the cyanobacteria harbored ancient versions of *sphR*, *sphS *and other phosphorus assimilation related genes that were regulated by the SphS/SphR system. Therefore, we conclude that the *sphR *and *sphS *genes in CCMP1375, CC9311 and CC9902 were lost during the course of evolution. Interestingly, CCMP1375 inhabits a lower layer of the sea water in oligotrophic open oceans [20], and both CC9311 [21] and CC9902 [22] were isolated from costal seawater, both of which are known to be relatively rich in P_i _[21,23]. It is likely that the abundant P_i _in these niches relieved the selection pressure to retain *sphR/sphR *genes in these organisms, and have resulted in their complete loss. Alternatively, the function of SphR could have been hitchhiked by another regulator, which resulted in its loss in these genomes. In any event, the loss of *sphR *would have in turn led to the degradation of otherwise conserved Pho boxes as it has been shown that the conservation of *cis*-regulatory binding sites is due to the constant purifying selection exerted from the binding interface of their *trans*-regulators [24].

### 2. Phosphorus assimilation pathways and their possible coupling to other biological pathways

Our analysis results indicate that cyanobacteria as a group of widely distributed microorganisms can utilize a broad range of phosphorus sources, including free P_i_, P_i_-containing organic compounds and C-P bond-containing phosphonates, as the relevant transporters and metabolic enzymes are found in their genomes. However, the distribution of these genes in each genome is quite different, reflecting the unique environment where a specific organism inhabits. The only genes that are ubiquitously encoded in all the 19 genomes are those that encode the four subunits of high affinity P_i _Pst uptake system, namely, the *pstS, pstC, pstA *and *pstB *genes, suggesting that P_i _is probably the most widely available source of phosphorus for cyanobacteria despite its low concentration in some environments. While many genes that are directly involved in the phosphorus assimilation pathway are predicted to be regulated by SphR, some others are not. This is in sharp contrast to their orthologues in *E. coli*, where all these genes are regulated by PhoB [8]. In addition, the regulatory modes of the phosphorus assimilation genes in cyanobacteria are also highly diversified in terms of the distribution of the predicted SphR binding sites for the orthologous genes, reflecting the heterogeneity and complexity of the environments where they inhabit.

Intriguingly, numerous putative new members of the Pho regulon are predicted in each of the analyzed genomes containing the *sphR *gene. Many of these appear to not be directly associated with phosphorus assimilation but instead encode proteins that are involved in photosynthesis, carbon fixation, nutrients/solutes transportation, signal transduction (sensor kinases), and transcription regulation (response regulators) (Table 2). A SphR binding site in the promoter region of these genes might serve as a mechanism of cross-talk between the phosphorus assimilation pathway and the pathways in which they play a direct role. It has in fact been shown that nitrogen fixation in IMS101 is restricted by phosphorus [3], an indication of coordination between the phosphorus assimilation and the nitrogen fixation. Interestingly, we have predicted several nitrogen assimilation genes to be putative members of the Pho regulon in several species/ecotypes (Table 2). In addition, it has been shown that in *E. coli *[25], *B. subtilis *[26], MED4 [17] and MIT9313 [17], P limitation is correlated with up- or down-regulations of both phosphate assimilation related proteins/genes and other proteins/genes. For instance, in *E. coli*, only 39 Pho regulon genes have been experimentally shown to be directly involved in phosphorus assimilation, while the number of proteins involved in the global response elicited by P limitation could be much larger as it has been shown previously that at least 413 genes are involved in the global response to P limitation in *E. coli *[25]. While some of these 413 genes might be under the direct control of PhoB, the others could be the results of cross-talks between the phosphorus assimilation and other biological pathways. This is probably achieved through a network of signal transduction proteins and transcriptional regulators that are directly or indirectly regulated by PhoB. Such a regulatory network is also likely to exist in cyanobacteria, as it was shown that P limitation results in changes in expression of 34 and 178 genes in MED4 and MIT9313, respectively [17], though only 5 and 2 of them are predicted to bear putative SphR binding sites, respectively. This suggests that the other genes are probably regulated by other regulators. Alternatively, these genes could be regulated by SphR through different binding motifs. In addition, it has been shown that the expression of the *urtA *gene in PCC6803 (*slr0447*) [12] and in MED4 (*PMM0970*) [17] is strongly repressed during phosphorus starvation. However, no *sphR *binding site has been experimentally identified in PCC6803 [12] or by our computational study. Thus the inhibition of *urtA *by P limitation is likely to be mediated by a different regulator other than SphR.

## Conclusion

Different cyanobacterial species/ecotypes encode diverse sets of genes responsible for the utilization of various sources of phosphorus available in their environment. Unlike in *E. coli*, only a portion of cyanobacterial genes that are directly involved in phosphorus assimilation are under the regulation of SphR in some species/ecotypes. In the three genomes, i.e., CCMP1375, CC9902 and CC9311, where the SphR gene is missing, the Pho boxes have degraded to a level that is indistinguishable from the randomly occurring ones (Figure 2). Thus, a regulator seems to play an important role in retaining its binding sites. In addition, we have also shown that the predicted Pho regulons in cyanobacteria might include genes known to play important roles in other biological process, such as photosynthesis, carbon fixation, nitrogen assimilation, signal transduction and transcription regulation. These genes might serve as bridging points to couple the phosphorus assimilation pathway to the pathways underlying these biological processes.

## Methods

### 1. Materials

The sequence and annotation files for the 19 sequenced cyanobacteria genomes were downloaded from the GenBank. These cyanobacterial genomes are *Anabaena variabilis ATCC 29413 *(ATCC29413), *Cyanobacteria bacterium Yellowstone A Prime *(Aprime), *Cyanobacteria bacterium Yellowstone B Prime *(Bprime), *Gloeobacter violaceus *PCC 7421 (PCC7421), *Nostoc sp*. PCC 7120 (PCC7120), *Prochlorococcus marinus *CCMP1375 (CCMP1375), *Prochlorococcus marinus *MED4 (MED4), *Prochlorococcus marinus *MIT 9312 (MIT9312), *Prochlorococcus marinus *MIT9313 (MIT9313), *Prochlorococcus marinus *NATL2A (NATL2A), *Synechococcus elongatus *PCC 7942 (PCC7942), *Synechococcus elongatus *PCC 6301 (PCC6301), *Synechococcus sp*. WH8102 (WH8102), *Synechococcus sp*. CC9605 (CC9605), *Synechococcus sp*. CC9902 (CC9902), *Synechococcus sp*. CC9311(CC9311), *Synechocystis sp*. PCC 6803 (PCC6803), *Thermosynechococcus elongates *BF-1(BF-1) and *Trichodesmium erythraeum IMS101 *(IMS101).

### 2. Prediction of operons

Multi-gene operons were predicted using our JPOP program [27,28] for each genome. For genes arranged in tandem on the same strand and not predicted to be part of an operon by JPOP, we considered them to form an operon if their intergenic distances are shorter than 45 base pairs. Genes that were not covered by this procedure were considered to each form a singleton operon.

### 3. Prediction of orthologs

We used the reciprocal best hit method [29] to predict orthologous genes between each two genomes by the BLASTP program with an E-value cutoff 10^-6 ^for both directions.

### 4. Phylogenetic footprinting

Since the operons *sphX-pstS1-C1-A1-B1-B1'*, *pstS2-C2-A2-B2*, and *phoA-nucH *in PCC6803 are known to be regulated by SphR through at least three tandem Pho boxes in their promoter regions, we identified the orthologues (if exist) of these genes in the other 18 genomes. We pooled the entire upstream regions (if a region is longer than 800 bases, only the immediate upstream 800 bases were used) of these orthologues in each genome according to the predicted operons. If an intergenic region is shorter than 100 bases, then 10 bases in its upstream coding region was included. Then a motif containing two 8 bp tandem repeats with a linker of 3 bp were searched in these sequences using the CUBIC program [15]. The identified motifs with a score above a pre-selected cutoff (p < 0.001) were returned. A profile was built for these identified two tandem sequences plus the linkers. A second profile was constructed by combining the two conserved 8 pb blocks in all these sequences.

### 5. Genome wide prediction of SphR binding sites

The two profiles of Pho boxes constructed above were used to scan the genome sequences for all possible SphR binding sites using an algorithm that we developed previously [13], which is briefly described as follows with some modifications.

For each predicted transcriptional unit *U*(*g*_1_,...,*g*_*n*_) containing genes *g*_1_, ..., *g*_*n *_in genome *G*, we extract the entire upstream intergenic region (if the region is longer than 800 bases, then only the immediate upstream 800 bases were extracted), denoted IU(g1,...,gn)
 MathType@MTEF@5@5@+=feaafiart1ev1aaatCvAUfKttLearuWrP9MDH5MBPbIqV92AaeXatLxBI9gBaebbnrfifHhDYfgasaacH8akY=wiFfYdH8Gipec8Eeeu0xXdbba9frFj0=OqFfea0dXdd9vqai=hGuQ8kuc9pgc9s8qqaq=dirpe0xb9q8qiLsFr0=vr0=vr0dc8meaabaqaciaacaGaaeqabaqabeGadaaakeaacqWGjbqsdaWgaaWcbaGaemyvauLaeiikaGIaem4zaC2aaSbaaWqaaiabigdaXaqabaWccqGGSaalcqGGUaGlcqGGUaGlcqGGUaGlcqGGSaalcqWGNbWzdaWgaaadbaGaemOBa4gabeaaliabcMcaPaqabaaaaa@3AB7@ for scanning for possible binding sites. If an intergenic region is shorter than 100 bases, then 10 bases in its upstream coding region was included. All the extracted IU(g1,...,gn)
 MathType@MTEF@5@5@+=feaafiart1ev1aaatCvAUfKttLearuWrP9MDH5MBPbIqV92AaeXatLxBI9gBaebbnrfifHhDYfgasaacH8akY=wiFfYdH8Gipec8Eeeu0xXdbba9frFj0=OqFfea0dXdd9vqai=hGuQ8kuc9pgc9s8qqaq=dirpe0xb9q8qiLsFr0=vr0=vr0dc8meaabaqaciaacaGaaeqabaqabeGadaaakeaacqWGjbqsdaWgaaWcbaGaemyvauLaeiikaGIaem4zaC2aaSbaaWqaaiabigdaXaqabaWccqGGSaalcqGGUaGlcqGGUaGlcqGGUaGlcqGGSaalcqWGNbWzdaWgaaadbaGaemOBa4gabeaaliabcMcaPaqabaaaaa@3AB7@'s in the genome are denoted as set *I*_*U*_. We also extracted a randomly chosen coding sequence with the same length as IU(g1,...,gn)
 MathType@MTEF@5@5@+=feaafiart1ev1aaatCvAUfKttLearuWrP9MDH5MBPbIqV92AaeXatLxBI9gBaebbnrfifHhDYfgasaacH8akY=wiFfYdH8Gipec8Eeeu0xXdbba9frFj0=OqFfea0dXdd9vqai=hGuQ8kuc9pgc9s8qqaq=dirpe0xb9q8qiLsFr0=vr0=vr0dc8meaabaqaciaacaGaaeqabaqabeGadaaakeaacqWGjbqsdaWgaaWcbaGaemyvauLaeiikaGIaem4zaC2aaSbaaWqaaiabigdaXaqabaWccqGGSaalcqGGUaGlcqGGUaGlcqGGUaGlcqGGSaalcqWGNbWzdaWgaaadbaGaemOBa4gabeaaliabcMcaPaqabaaaaa@3AB7@ from *G*, denoted as CU(g1,...,gn)
 MathType@MTEF@5@5@+=feaafiart1ev1aaatCvAUfKttLearuWrP9MDH5MBPbIqV92AaeXatLxBI9gBaebbnrfifHhDYfgasaacH8akY=wiFfYdH8Gipec8Eeeu0xXdbba9frFj0=OqFfea0dXdd9vqai=hGuQ8kuc9pgc9s8qqaq=dirpe0xb9q8qiLsFr0=vr0=vr0dc8meaabaqaciaacaGaaeqabaqabeGadaaakeaacqWGdbWqdaWgaaWcbaGaemyvauLaeiikaGIaem4zaC2aaSbaaWqaaiabigdaXaqabaWccqGGSaalcqGGUaGlcqGGUaGlcqGGUaGlcqGGSaalcqWGNbWzdaWgaaadbaGaemOBa4gabeaaliabcMcaPaqabaaaaa@3AAB@ for scanning for randomly occurring motifs. All the extracted CU(g1,...,gn)
 MathType@MTEF@5@5@+=feaafiart1ev1aaatCvAUfKttLearuWrP9MDH5MBPbIqV92AaeXatLxBI9gBaebbnrfifHhDYfgasaacH8akY=wiFfYdH8Gipec8Eeeu0xXdbba9frFj0=OqFfea0dXdd9vqai=hGuQ8kuc9pgc9s8qqaq=dirpe0xb9q8qiLsFr0=vr0=vr0dc8meaabaqaciaacaGaaeqabaqabeGadaaakeaacqWGdbWqdaWgaaWcbaGaemyvauLaeiikaGIaem4zaC2aaSbaaWqaaiabigdaXaqabaWccqGGSaalcqGGUaGlcqGGUaGlcqGGUaGlcqGGSaalcqWGNbWzdaWgaaadbaGaemOBa4gabeaaliabcMcaPaqabaaaaa@3AAB@'s in the genome are denoted as set *C*_*U*_. We say that IU(g1,...,gn)
 MathType@MTEF@5@5@+=feaafiart1ev1aaatCvAUfKttLearuWrP9MDH5MBPbIqV92AaeXatLxBI9gBaebbnrfifHhDYfgasaacH8akY=wiFfYdH8Gipec8Eeeu0xXdbba9frFj0=OqFfea0dXdd9vqai=hGuQ8kuc9pgc9s8qqaq=dirpe0xb9q8qiLsFr0=vr0=vr0dc8meaabaqaciaacaGaaeqabaqabeGadaaakeaacqWGjbqsdaWgaaWcbaGaemyvauLaeiikaGIaem4zaC2aaSbaaWqaaiabigdaXaqabaWccqGGSaalcqGGUaGlcqGGUaGlcqGGUaGlcqGGSaalcqWGNbWzdaWgaaadbaGaemOBa4gabeaaliabcMcaPaqabaaaaa@3AB7@ and CU(g1,...,gn)
 MathType@MTEF@5@5@+=feaafiart1ev1aaatCvAUfKttLearuWrP9MDH5MBPbIqV92AaeXatLxBI9gBaebbnrfifHhDYfgasaacH8akY=wiFfYdH8Gipec8Eeeu0xXdbba9frFj0=OqFfea0dXdd9vqai=hGuQ8kuc9pgc9s8qqaq=dirpe0xb9q8qiLsFr0=vr0=vr0dc8meaabaqaciaacaGaaeqabaqabeGadaaakeaacqWGdbWqdaWgaaWcbaGaemyvauLaeiikaGIaem4zaC2aaSbaaWqaaiabigdaXaqabaWccqGGSaalcqGGUaGlcqGGUaGlcqGGUaGlcqGGSaalcqWGNbWzdaWgaaadbaGaemOBa4gabeaaliabcMcaPaqabaaaaa@3AAB@ are associated with *U*(*g*_1_,...,*g*_*n*_) and with each of the genes *g*_1_, ..., and *g*_*n *_as well. The score of a putative binding site found in an extracted sequence *t *(*t *∈ *I*_*U *_*or t *∈ *C*_*U*_) by scanning with a profile *M *is defined as

sM(t)=max⁡h⊂t∑i=1lIiln⁡p(i,h(i))q(h(i)),
 MathType@MTEF@5@5@+=feaafiart1ev1aaatCvAUfKttLearuWrP9MDH5MBPbIqV92AaeXatLxBI9gBaebbnrfifHhDYfgasaacH8akY=wiFfYdH8Gipec8Eeeu0xXdbba9frFj0=OqFfea0dXdd9vqai=hGuQ8kuc9pgc9s8qqaq=dirpe0xb9q8qiLsFr0=vr0=vr0dc8meaabaqaciaacaGaaeqabaqabeGadaaakeaacqWGZbWCdaWgaaWcbaGaemyta0eabeaakiabcIcaOiabdsha0jabcMcaPiabg2da9maaxababaGagiyBa0MaeiyyaeMaeiiEaGhaleaacqWGObaAcqGHckcZcqWG0baDaeqaaOWaaabCaeaacqWGjbqsdaWgaaWcbaGaemyAaKgabeaaaeaacqWGPbqAcqGH9aqpcqaIXaqmaeaacqWGSbaBa0GaeyyeIuoakiGbcYgaSjabc6gaUnaalaaabaGaemiCaaNaeiikaGIaemyAaKMaeiilaWIaemiAaGMaeiikaGIaemyAaKMaeiykaKIaeiykaKcabaGaemyCaeNaeiikaGIaemiAaGMaeiikaGIaemyAaKMaeiykaKIaeiykaKcaaiabcYcaSaaa@5B56@

Ii=(∑b∈{A,C,G,T}p(i,b)ln⁡p(i,b)q(b))/a,
 MathType@MTEF@5@5@+=feaafiart1ev1aaatCvAUfKttLearuWrP9MDH5MBPbIqV92AaeXatLxBI9gBaebbnrfifHhDYfgasaacH8akY=wiFfYdH8Gipec8Eeeu0xXdbba9frFj0=OqFfea0dXdd9vqai=hGuQ8kuc9pgc9s8qqaq=dirpe0xb9q8qiLsFr0=vr0=vr0dc8meaabaqaciaacaGaaeqabaqabeGadaaakeaacqWGjbqsdaWgaaWcbaGaemyAaKgabeaakiabg2da9iabcIcaOmaaqafabaGaemiCaaNaeiikaGIaemyAaKMaeiilaWIaemOyaiMaeiykaKIagiiBaWMaeiOBa42aaSaaaeaacqWGWbaCcqGGOaakcqWGPbqAcqGGSaalcqWGIbGycqGGPaqkaeaacqWGXbqCcqGGOaakcqWGIbGycqGGPaqkaaGaeiykaKIaei4la8IaemyyaegaleaacqWGIbGycqGHiiIZcqGG7bWEcqWGbbqqcqGGSaalcqWGdbWqcqGGSaalcqWGhbWrcqGGSaalcqWGubavcqGG9bqFaeqaniabggHiLdGccqGGSaalaaa@58A7@

a=n+1n+4ln⁡(n+1)−ln⁡(n+4)−1n+4∑b∈{A,C,G,T}ln⁡q(b)−nn+4ln⁡min⁡b∈{A,C,G,T}q(b),
 MathType@MTEF@5@5@+=feaafiart1ev1aaatCvAUfKttLearuWrP9MDH5MBPbIqV92AaeXatLxBI9gBaebbnrfifHhDYfgasaacH8akY=wiFfYdH8Gipec8Eeeu0xXdbba9frFj0=OqFfea0dXdd9vqai=hGuQ8kuc9pgc9s8qqaq=dirpe0xb9q8qiLsFr0=vr0=vr0dc8meaabaqaciaacaGaaeqabaqabeGadaaakeaacqWGHbqycqGH9aqpdaWcaaqaaiabd6gaUjabgUcaRiabigdaXaqaaiabd6gaUjabgUcaRiabisda0aaacyGGSbaBcqGGUbGBcqGGOaakcqWGUbGBcqGHRaWkcqaIXaqmcqGGPaqkcqGHsislcyGGSbaBcqGGUbGBcqGGOaakcqWGUbGBcqGHRaWkcqaI0aancqGGPaqkcqGHsisldaWcaaqaaiabigdaXaqaaiabd6gaUjabgUcaRiabisda0aaadaaeqbqaaiGbcYgaSjabc6gaUjabdghaXjabcIcaOiabdkgaIjabcMcaPiabgkHiTaWcbaGaemOyaiMaeyicI4Saei4EaSNaemyqaeKaeiilaWIaem4qamKaeiilaWIaem4raCKaeiilaWIaemivaqLaeiyFa0habeqdcqGHris5aOWaaSaaaeaacqWGUbGBaeaacqWGUbGBcqGHRaWkcqaI0aanaaGagiiBaWMaeiOBa42aaCbeaeaacyGGTbqBcqGGPbqAcqGGUbGBaSqaaiabdkgaIjabgIGiolabcUha7jabdgeabjabcYcaSiabdoeadjabcYcaSiabdEeahjabcYcaSiabdsfaujabc2ha9bqabaGccqWGXbqCcqGGOaakcqWGIbGycqGGPaqkcqGGSaalaaa@8013@

where *l *is the length of the binding sites of *M, h *any substring of *t *with length *l*, *h(i) *the base at position *i *of *h*, *p(i,b) *the relative frequency of base *b *at position *i *in *M*, *q(b) *the relative frequency of base *b *occurring in the background, and *n *the number of sequences in *M*. A pseudo-count 1 is added to the frequency of each base at each position in the profile when computing *p(i,b)*. The coefficient *a *is used for normalization so that *I*_*i *_is in the region [0,1].

When multiple profiles *M*_1_, ..., *M*_*z *_are used for scanning, the score of concurrence of multiple putative binding sites in the sequence *t *is defined as

sM1...Mz(t)=∑j=1zsMj(t).
 MathType@MTEF@5@5@+=feaafiart1ev1aaatCvAUfKttLearuWrP9MDH5MBPbIqV92AaeXatLxBI9gBaebbnrfifHhDYfgasaacH8akY=wiFfYdH8Gipec8Eeeu0xXdbba9frFj0=OqFfea0dXdd9vqai=hGuQ8kuc9pgc9s8qqaq=dirpe0xb9q8qiLsFr0=vr0=vr0dc8meaabaqaciaacaGaaeqabaqabeGadaaakeaacqWGZbWCdaWgaaWcbaGaemyta00aaSbaaWqaaiabigdaXaqabaWccqGGUaGlcqGGUaGlcqGGUaGlcqWGnbqtdaWgaaadbaGaemOEaOhabeaaaSqabaGccqGGOaakcqWG0baDcqGGPaqkcqGH9aqpdaaeWbqaaiabdohaZnaaBaaaleaacqWGnbqtdaWgaaadbaGaemOAaOgabeaaaSqabaGccqGGOaakcqWG0baDcqGGPaqkaSqaaiabdQgaQjabg2da9iabigdaXaqaaiabdQha6bqdcqGHris5aOGaeiOla4caaa@49C9@

We now define a score for concurrence of multiple binding sites in a sequence associated with a transcription unit by also considering the presence of similar motifs in the sequences associated with its orthologous genes in other genomes. Let *t *be the extracted sequence (*t *∈ *I*_*U *_*or t *∈ *C*_*U*_) associated with a transcription unit *U*(*g*_1_...*g*_*n*_) in genome *T*. If *g*_*i *_has orthologues in *m*_*i *_closely related genomes *G*_1_,...,*G*_*mi*_, let *o*_*k*_(*g*_*i*_) be the same type of extracted sequence (*o*_*k*_(*g*_*i*_) ∈ *I*_*U *_or *o*_*k*_(*g*_*i*_) ∈ *C*_*U*_) associated with the orthologue of *g*_*i *_in genome *G*_*k*_. Then the score of concurrence of the multiple binding sites in *t *is redefined as

s(t)=sM1...Mz(t)+max⁡1<i<n∑j=1z∑k=1milj−di,j,kmiljsMj(ok(gi)),
 MathType@MTEF@5@5@+=feaafiart1ev1aaatCvAUfKttLearuWrP9MDH5MBPbIqV92AaeXatLxBI9gBaebbnrfifHhDYfgasaacH8akY=wiFfYdH8Gipec8Eeeu0xXdbba9frFj0=OqFfea0dXdd9vqai=hGuQ8kuc9pgc9s8qqaq=dirpe0xb9q8qiLsFr0=vr0=vr0dc8meaabaqaciaacaGaaeqabaqabeGadaaakeaacqWGZbWCcqGGOaakcqWG0baDcqGGPaqkcqGH9aqpcqWGZbWCdaWgaaWcbaGaemyta00aaSbaaWqaaiabigdaXaqabaWccqGGUaGlcqGGUaGlcqGGUaGlcqWGnbqtdaWgaaadbaGaemOEaOhabeaaaSqabaGccqGGOaakcqWG0baDcqGGPaqkcqGHRaWkdaWfqaqaaiGbc2gaTjabcggaHjabcIha4bWcbaGaeGymaeJaeyipaWJaemyAaKMaeyipaWJaemOBa4gabeaakmaaqahabaWaaabCaeaadaWcaaqaaiabdYgaSnaaBaaaleaacqWGQbGAaeqaaOGaeyOeI0Iaemizaq2aaSbaaSqaaiabdMgaPjabcYcaSiabdQgaQjabcYcaSiabdUgaRbqabaaakeaacqWGTbqBdaWgaaWcbaGaemyAaKgabeaakiabdYgaSnaaBaaaleaacqWGQbGAaeqaaaaakiabdohaZnaaBaaaleaacqWGnbqtdaWgaaadbaGaemOAaOgabeaaaSqabaGccqGGOaakcqWGVbWBdaWgaaWcbaGaem4AaSgabeaakiabcIcaOiabdEgaNnaaBaaaleaacqWGPbqAaeqaaOGaeiykaKcaleaacqWGRbWAcqGH9aqpcqaIXaqmaeaacqWGTbqBdaWgaaadbaGaemyAaKgabeaaa0GaeyyeIuoakiabcMcaPaWcbaGaemOAaOMaeyypa0JaeGymaedabaGaemOEaOhaniabggHiLdGccqGGSaalaaa@7942@

where *d*_*i*,,*j*,*k*, _is the Hamming distance between the sequence found by using profile *M*_*j *_in *t*, and the corresponding sequence found in *o*_*k*_*(g*_*i*_*)*, and *l*_*j *_is the length of the sequences of profile *M*_*j*_. If a group of genomes are highly similar to one another in their sequences, then the orthologues information among them will not be used for their respective binding site predictions to avoid counting non-functionally conserved sequences. In the present study, such groups are PCC6801 and PCC7942 as well as Aprime and Bprime.

When we consider all the extracted sequences in *I*_*U *_and *C*_*U *_in a genome, the scores of binding sites found in *v *∈ *I*_*U *_and *w *∈ *C*_*U*_, SIU
 MathType@MTEF@5@5@+=feaafiart1ev1aaatCvAUfKttLearuWrP9MDH5MBPbIqV92AaeXatLxBI9gBaebbnrfifHhDYfgasaacH8akY=wiFfYdH8Gipec8Eeeu0xXdbba9frFj0=OqFfea0dXdd9vqai=hGuQ8kuc9pgc9s8qqaq=dirpe0xb9q8qiLsFr0=vr0=vr0dc8meaabaqaciaacaGaaeqabaqabeGadaaakeaacqWGtbWudaWgaaWcbaGaemysaK0aaSbaaWqaaiabdwfavbqabaaaleqaaaaa@308D@ ∈ *s*(*v*) and SCU
 MathType@MTEF@5@5@+=feaafiart1ev1aaatCvAUfKttLearuWrP9MDH5MBPbIqV92AaeXatLxBI9gBaebbnrfifHhDYfgasaacH8akY=wiFfYdH8Gipec8Eeeu0xXdbba9frFj0=OqFfea0dXdd9vqai=hGuQ8kuc9pgc9s8qqaq=dirpe0xb9q8qiLsFr0=vr0=vr0dc8meaabaqaciaacaGaaeqabaqabeGadaaakeaacqWGtbWudaWgaaWcbaGaem4qam0aaSbaaWqaaiabdwfavbqabaaaleqaaaaa@3081@ ∈ *s*(*w*), respectively, are random variables. Let *p*(SIU
 MathType@MTEF@5@5@+=feaafiart1ev1aaatCvAUfKttLearuWrP9MDH5MBPbIqV92AaeXatLxBI9gBaebbnrfifHhDYfgasaacH8akY=wiFfYdH8Gipec8Eeeu0xXdbba9frFj0=OqFfea0dXdd9vqai=hGuQ8kuc9pgc9s8qqaq=dirpe0xb9q8qiLsFr0=vr0=vr0dc8meaabaqaciaacaGaaeqabaqabeGadaaakeaacqWGtbWudaWgaaWcbaGaemysaK0aaSbaaWqaaiabdwfavbqabaaaleqaaaaa@308D@ > *s*) and *p*(SCU
 MathType@MTEF@5@5@+=feaafiart1ev1aaatCvAUfKttLearuWrP9MDH5MBPbIqV92AaeXatLxBI9gBaebbnrfifHhDYfgasaacH8akY=wiFfYdH8Gipec8Eeeu0xXdbba9frFj0=OqFfea0dXdd9vqai=hGuQ8kuc9pgc9s8qqaq=dirpe0xb9q8qiLsFr0=vr0=vr0dc8meaabaqaciaacaGaaeqabaqabeGadaaakeaacqWGtbWudaWgaaWcbaGaem4qam0aaSbaaWqaaiabdwfavbqabaaaleqaaaaa@3081@ > *s*) be the cumulative probability that *I*_*U *_and *C*_*U *_have putative binding sites with scores *s*(*v*)>*s *and *s*(*w*)>*s*, respectively. To compute *p*(SCU
 MathType@MTEF@5@5@+=feaafiart1ev1aaatCvAUfKttLearuWrP9MDH5MBPbIqV92AaeXatLxBI9gBaebbnrfifHhDYfgasaacH8akY=wiFfYdH8Gipec8Eeeu0xXdbba9frFj0=OqFfea0dXdd9vqai=hGuQ8kuc9pgc9s8qqaq=dirpe0xb9q8qiLsFr0=vr0=vr0dc8meaabaqaciaacaGaaeqabaqabeGadaaakeaacqWGtbWudaWgaaWcbaGaem4qam0aaSbaaWqaaiabdwfavbqabaaaleqaaaaa@3081@ > *s*) for a genome, we generated 300 CU(g1,...,gn)
 MathType@MTEF@5@5@+=feaafiart1ev1aaatCvAUfKttLearuWrP9MDH5MBPbIqV92AaeXatLxBI9gBaebbnrfifHhDYfgasaacH8akY=wiFfYdH8Gipec8Eeeu0xXdbba9frFj0=OqFfea0dXdd9vqai=hGuQ8kuc9pgc9s8qqaq=dirpe0xb9q8qiLsFr0=vr0=vr0dc8meaabaqaciaacaGaaeqabaqabeGadaaakeaacqWGdbWqdaWgaaWcbaGaemyvauLaeiikaGIaem4zaC2aaSbaaWqaaiabigdaXaqabaWccqGGSaalcqGGUaGlcqGGUaGlcqGGUaGlcqGGSaalcqWGNbWzdaWgaaadbaGaemOBa4gabeaaliabcMcaPaqabaaaaa@3AAB@'s associated with each transcription unit *U*(*g*_1_,...,*g*_*n*_) in each genome and computed the score for each CU(g1,...,gn)
 MathType@MTEF@5@5@+=feaafiart1ev1aaatCvAUfKttLearuWrP9MDH5MBPbIqV92AaeXatLxBI9gBaebbnrfifHhDYfgasaacH8akY=wiFfYdH8Gipec8Eeeu0xXdbba9frFj0=OqFfea0dXdd9vqai=hGuQ8kuc9pgc9s8qqaq=dirpe0xb9q8qiLsFr0=vr0=vr0dc8meaabaqaciaacaGaaeqabaqabeGadaaakeaacqWGdbWqdaWgaaWcbaGaemyvauLaeiikaGIaem4zaC2aaSbaaWqaaiabigdaXaqabaWccqGGSaalcqGGUaGlcqGGUaGlcqGGUaGlcqGGSaalcqWGNbWzdaWgaaadbaGaemOBa4gabeaaliabcMcaPaqabaaaaa@3AAB@ to avoid possible biased sampling. We then used the following log-odds ratio (*LOR*) function to estimate the confidence of the predictions in a genome,

LOR(s)=ln⁡p(SIU>s)p(SCU>s).
 MathType@MTEF@5@5@+=feaafiart1ev1aaatCvAUfKttLearuWrP9MDH5MBPbIqV92AaeXatLxBI9gBaebbnrfifHhDYfgasaacH8akY=wiFfYdH8Gipec8Eeeu0xXdbba9frFj0=OqFfea0dXdd9vqai=hGuQ8kuc9pgc9s8qqaq=dirpe0xb9q8qiLsFr0=vr0=vr0dc8meaabaqaciaacaGaaeqabaqabeGadaaakeaacqWGmbatcqWGpbWtcqWGsbGucqGGOaakcqWGZbWCcqGGPaqkcqGH9aqpcyGGSbaBcqGGUbGBdaWcaaqaaiabdchaWjabcIcaOiabdofatnaaBaaaleaacqWGjbqsdaWgaaadbaGaemyvaufabeaaaSqabaGccqGH+aGpcqWGZbWCcqGGPaqkaeaacqWGWbaCcqGGOaakcqWGtbWudaWgaaWcbaGaem4qam0aaSbaaWqaaiabdwfavbqabaaaleqaaOGaeyOpa4Jaem4CamNaeiykaKcaaiabc6caUaaa@4AF0@

Since *p*(SCU
 MathType@MTEF@5@5@+=feaafiart1ev1aaatCvAUfKttLearuWrP9MDH5MBPbIqV92AaeXatLxBI9gBaebbnrfifHhDYfgasaacH8akY=wiFfYdH8Gipec8Eeeu0xXdbba9frFj0=OqFfea0dXdd9vqai=hGuQ8kuc9pgc9s8qqaq=dirpe0xb9q8qiLsFr0=vr0=vr0dc8meaabaqaciaacaGaaeqabaqabeGadaaakeaacqWGtbWudaWgaaWcbaGaem4qam0aaSbaaWqaaiabdwfavbqabaaaleqaaaaa@3081@ > *s*) is the probability of type I error for testing the null hypothesis that *I*_*U *_does not contain a binding site when SIU
 MathType@MTEF@5@5@+=feaafiart1ev1aaatCvAUfKttLearuWrP9MDH5MBPbIqV92AaeXatLxBI9gBaebbnrfifHhDYfgasaacH8akY=wiFfYdH8Gipec8Eeeu0xXdbba9frFj0=OqFfea0dXdd9vqai=hGuQ8kuc9pgc9s8qqaq=dirpe0xb9q8qiLsFr0=vr0=vr0dc8meaabaqaciaacaGaaeqabaqabeGadaaakeaacqWGtbWudaWgaaWcbaGaemysaK0aaSbaaWqaaiabdwfavbqabaaaleqaaaaa@308D@ is greater than a cutoff *s*_*c*_, we used it to estimate the false positive rate of our prediction, *i.e*., the *p*-value, and a cutoff 0.01 was used for the SphR binding site prediction in each genome.

## Abbreviations

P_i_, inorganic phosphate; LOR, log odds ratio; ATCC29413, *Anabaena variabilis *ATCC 29413; Aprime, *Cyanobacteria bacterium *Yellowstone A Prime; Bprime, *Cyanobacteria bacterium *Yellowstone B Prime; PCC7421, *Gloeobacter violaceus *PCC 7421; PCC7120, *Nostoc sp*. PCC 7120; CCMP1375, *Prochlorococcus marinus *CCMP1375; MED4, *Prochlorococcus marinus *MED4; MIT9312, *Prochlorococcus marinus *MIT 9312; MIT9313, *Prochlorococcus marinus *MIT9313; NATL2A, *Prochlorococcus marinus *NATL2A; PCC7942, *Synechococcus elongatus *PCC 7942; PCC6301, *Synechococcus elongatus *PCC 6301; WH8102, *Synechococcus sp*. WH8102; CC9605, *Synechococcus sp*. CC9605; CC9902, *Synechococcus sp*. CC9902; CC9311, *Synechococcus sp*. CC9311; PCC6803, *Synechocystis sp*. PCC 6803; BF-1, *Thermosynechococcus elongates BF-1*; and IMS101, *Trichodesmium erythraeum *IMS10.

## Authors' contributions

ZS designed and conducted the experiments, and wrote the manuscript; VO designed and tested a scoring function, and implemented a part of the program; YX conceived the project and wrote the manuscript. All authors read and approved the final manuscript.

## Supplementary Material

Additional file 1 Predicted Pho boxes in 16 cyanobacterial genomes. Tables list the predicted SphR binding sites in 16 cyanobacterial genomes that encode the *sphR *gene. Click here for file
