# Supplementary material for: Computational prediction of Pho regulons in cyanobacteria
Source: BMC Genomics. 2007 Jun 8;8:156. doi: 10.1186/1471-2164-8-156 (PMC1906773; doi:10.1186/1471-2164-8-156)
Supplement: Additional file 1 — Predicted Pho boxes in 16 cyanobacterial genomes. Tables list the predicted SphR binding sites in 16 cyanobacterial genomes that encode the sphR gene. [file 1471-2164-8-156-S1.pdf]

Table 1s. Predicted Pho boxes for ATCC29413

Rank	Transcription unit	Names	Pho Boxes	Position	Score	Annotation of the first gene in the transcription unit
1	*Ava_3741*	-	ATTAATAActtCTTAAGCCttaATTAAACG	-102	10.3	hypothetical protein
2	*Ava_1501*	-	ATTAAATTaatCTTTAACTggtCTTAACCT	-318	9.97	hypothetical protein
3	*Ava_3295*	-	TTTAATTTgttATTATCCTtccTTTAATCA	-171	9.32	hypothetical protein
4	*Ava_3265*	-	ATTAAACTttaATTAACTTctaGTTAATGG	-80	9.29	Putative Ig
5	*Ava_2944*	-	ATTATGATcaaGTTATAAAaacTTTAAACT	-234	9.12	Multi-sensor Signal Transduction Histidine Kinase
6	*Ava_4726*	-	ATTAATCTtttAATAATCTctcGATAAACA	-116	9.11	Na-Ca exchanger/integrin-beta4
7	*Ava_2541*	*phoA*	GTTAATCTtcaGTTAACCTaaaTTTATATT	-111	9.07	alkaline phosphatase-like
8	*Ava_2252*	-	CTTAACATcgtCTTAAACCctgCTTGATGA	-92	8.98	hypothetical protein
9	*Ava_2176*	-	TTTTGCCTtggGTTAAGAGgaaCTTAACCT	-123	8.96	hypothetical protein
10	*Ava_2887 Ava_2886 Ava_2885 Ava_2884*	- - - -	TTTAACATaatGTTAAGATattGTTAACTA	-159	8.81	hypothetical protein
11	*Ava_4516 Ava_4515 Ava_4514 Ava_4513 Ava_4512*	*pstS2 pstC2 pstA2 pstB2*	TATTTGTAtatTTTAACTTgaaATTAACCA	-403	8.75	hypothetical protein
12	*Ava_5066 Ava_5065*	- -	TTTAATGTataCTTAACCTattGTTAACCT	-38	8.74	hypothetical protein
13	*Ava_3388*	-	ATTTTGCTattATTAATCTctaGATAATCT	-487	8.68	PEP-utilizing enzyme
14	*Ava_1819*	-	GTTAAGGCttaTTTAAAGTtcgGTTAATTA	-42	8.67	hypothetical protein
15	*Ava_4000*	-	GTTTGCAActtTTTAACCTctgCTTATACA	-95	8.62	hypothetical protein
16	*Ava_2065*	-	ATTAATCAataTATAACCTgacGTTAACAC	-239	8.62	Twin-arginine translocation pathway signal
17	*Ava_2477 Ava_2478 Ava_2479 Ava_2480*	*pstS1 pstC1, pstA1, pstB1'*	TTTTTCCTtgtTTTAACCTttcGTTAACCT	-66	8.54	pstS1
18	*Ava_1429*	-	TTTTAGGAagcTTTAAATAcaaCTTACCTA	-126	8.48	Glycoside hydrolase, starch-binding
19	*Ava_2324*	-	TTTAACTAgagCTTAAAGCaacTTTGAAGT	-128	8.45	Two Component Transcriptional Regulator, LuxR family
20	*Ava_2432*	-	GTTAAACTggcATTAATTTacaGTTAAGAA	-290	8.41	hypothetical protein
21	*Ava_3667*	-	TTTAATGAaagCTTTCACCgcaATTTCAAG	-252	8.4	hypothetical protein
22	*Ava_0558*	-	ATTAGTATcaaCTTCTACTtcaCTTAAAGC	-81	8.39	hypothetical protein
23	*Ava_2500*	-	TGTAATTTctaCTTGAATGcgtCTTTATCA	-165	8.37	Rieske (2Fe-2S) region
24	*Ava_0990*	-	ATTAACTAataATTAAATAactCTTAAAGG	-79	8.35	hypothetical protein
25	*Ava_0892 Ava_0891 Ava_0890*	- - -	ATTTAAAAactCTTTATATtttATTAGTAA	-186	8.35	hypothetical protein
26	*Ava_4129*	-	TTTTACCTctcATTAAATTtgaCTTAAACT	-196	8.3	hypothetical protein
27	*Ava_3716*	*sphX*	CTTTACTGataCATCAACTtttTTTGATGA	-104	8.29	hypothetical protein, sphX
28	*Ava_4785*	-	TATTAAAAttgCTTAACACttcGTTACACT	-61	8.28	Peptidase M50
29	*Ava_2699*	-	TTTCACCTtaaATTAAGGTttaCTTAAACT	-171	8.28	hypothetical protein
30	*Ava_0064*	-	TTTAAAGAattTTTGCGCCtgaCTTTACCT	-480	8.28	Signal Transduction Histidine Kinase
31	*Ava_2230 Ava_2231*	- -	ATTATACAtaaCTTAGTCAagaCTTAGGTT	-162	8.26	hypothetical protein
32	*Ava_2778*	-	ATTTTGGGaatCTTCATATattTTTACACT	-522	8.26	Integrins alpha chain
33	*Ava_4727*	-	GTTCTTTTgaaCTTATTTTtttCTTAATCT	-122	8.17	Glycerophosphoryl diester phosphodiesterase
34	*Ava_0482 Ava_0481*	- -	CTTGAAAAaatCATCAACAaaaATTAGCAA	-82	8.17	hypothetical protein
35	*Ava_3592 Ava_3593*	- -	CTTGACATtccATTTTGCTaatCTTATAAC	-38	8.17	Protein of unknown function DUF262
36	*Ava_0968 Ava_0967*	- -	GTTAACAAtagGTTGAACAtatTTTAAGGG	-21	8.14	hypothetical protein
37	*Ava_2431 Ava_2430*	- -	CTTAACTGtaaATTAATGCcagTTTAACTA	-246	8.12	FAD-dependent pyridine nucleotide-disulphide oxidoreductase
38	*Ava_3624 Ava_3623*	- -	CTTACCTAaaaCCTAAGCTgagGTTGGTGT	-123	8.12	hypothetical protein
39	*Ava_4098 Ava_4097*	- -	ATTTTGGAcgcATTGATCAtagCTTCAGCT	-197	8.1	hypothetical protein
40	*Ava_0548*	-	TTTTACGCcatGATACTCTgatTTTTACTA	-510	8.1	hypothetical protein
41	*Ava_2832 Ava_2831*	- -	TTTAACAGtggATTCAAATctaCGTGATAG	-1	8.1	Esterase/lipase/thioesterase
42	*Ava_0785*	-	TTTATCTGaatATTAAAGCtatCTTAAGCC	-474	8.1	hypothetical protein
43	*Ava_1820*	-	ATTAACCGaacTTTAAATAagcCTTAACTG	-136	8.08	Pseudouridine synthase, Rsu
44	*Ava_1370*	-	ATTAAACGtgaATTCGACGgaaCCTAAACC	-352	8.07	hypothetical protein
45	*Ava_4986*	-	ATTAATCTtttCTTTATCTtctCTCCATTC	-650	8.06	hypothetical protein
46	*Ava_3968*	-	TTTTACTTtttCTTTGTCAcatTCTATTAA	-69	8.06	hypothetical protein
47	*Ava_4728*	*nucH*	TTTTACCTtttCCTATTATtctCTTAATCT	-174	8.04	Endonuclease/exonuclease/phosphatase
48	*Ava_2698*	-	TTAAATCTaagTTTAAGTAaacCTTAATTT	-158	8.04	hypothetical protein
49	*Ava_3614*	-	CTTTCAATcagTTTGTCATattTTTAAGCT	-36	8.04	Oxidoreductase, molybdopterin binding
50	*Ava_4202*	-	GTTAAATTagaGTTAAAGTcggCTTTTCCC	-11	8.01	Protein of unknown function DUF820
51	*Ava_4122 Ava_4123 Ava_4124*	- - -	ATTAACAAaatATTCAACGcatCTTAAAAG	-470	8.01	hypothetical protein
52	*Ava_2524*	-	CTTAATTAaatCTTAATAAtaaATTTCAAT	-238	7.99	Multi-sensor Signal Transduction Histidine Kinase
53	*Ava_1100*	-	TTTGTTCAtaaCTTCAATGcacTTTATACA	-282	7.99	hypothetical protein
54	*Ava_0789*	-	AATAAGGCataTTTAAGCGataCATAGCAT	-19	7.98	hypothetical protein

Table 2s. Predicted Pho boxes for Aprime

Rank	Transcription unit	Names	Pho boxes	Position	Score	Annotation of the first gene in the transcription unit
1	*CYA_1732 CYA_1733 CYA_1734 CYA_1735 CYA_1736*	*pstS pstC pstA pstB* -	TTTTCTCTtgaCTTAACCCaaaGTTAACCT	-69	11.04	phosphate ABC transporter, phosphate-binding protein
2	*CYA_1541*	-	CTTAACCTgggCATAACCTctcCTTTACTT	-153	10.28	transcriptional regulator, Crp/Fnr family
3	*CYA_1552 CYA_1553 CYA_1554 CYA_1555 CYA_1556 CYA_1557*	*pstS pstC pstA pstB* -	AATAACCTgaaTTTAACCTcttGTTAACCA	-175	10.11	phosphate ABC transporter, phosphate-binding protein
4	*CYA_1696*	-	CTTAACCTgggCTTGGTCTctcCTTAACTT	-135	10.10	hypothetical protein
5	*CYA_0900 CYA_0899*	- -	CTTATATCtggTTTAAAGTggcCATAGTTT	-158	9.59	nucleotidyltransferase domain protein
6	*CYA_2344 CYA_2343*	- -	CTTTATGCcccGTTAACTCtaaTTTAACCT	-169	9.50	hypothetical protein
7	*CYA_2183*	-	GTTAAGTTcaaGCTAAAGTtcaATTGTTCT	-246	9.21	starch binding domain protein
8	*CYA_2382 CYA_2383 CYA_2385*	*chlL* - *chlN*	CTTTAACCtgtTTTAGACTtccCTTAACTC	-69	9.15	light-independent protochlorophyllide reductase, iron-sulfur ATP-binding protein
9	*CYA_1551 CYA_1550 CYA_1549 CYA_1548*	*rplI dnaB* - -	GTTAACAAgagGTTAAATTcagGTTATTTC	-141	8.92	ribosomal protein L9
10	*CYA_0061 CYA_0060*	- -	CTTCTGTActtCTTCAAGTaggGTTGACCT	-114	8.92	RNA polymerase sigma-70 factor family
11	*CYA_1316*	-	CTTTTGAGcatTTTTAGAAacgCTTAAGCT	-330	8.83	DNA internalization competence protein, ComEC/Rec2 family
12	*CYA_1625*	*eda*	TTTTAATTgcaATTAAACTtaaAATCAACC	-51	8.73	2-dehydro-3-deoxyphosphogluconate aldolase/4-hydroxy-2-oxoglutarate aldolase
13	*CYA_2759*	-	TTTCAGCAaatCTTAGCCGcctCCTTAGCC	-164	8.73	cation transporter, P-type ATPase family
14	*CYA_0861 CYA_0863*	- *proC*	GTTAAATTgtgATTAAATTttgTGTAAAGC	-533	8.72	hypothetical protein
15	*CYA_0781*	-	GTTATGAGacaGTTAAGAGataGTTAACGT	-157	8.71	alkaline phosphatase
16	*CYA_2534 CYA_2533 CYA_2532 CYA_2531 CYA_2529*	- - - - -	TTTGAGCGactGATAAACTcctCTTTTTAG	-595	8.70	cyclopropane-fatty-acyl-phospholipid synthase
17	*CYA_0220 CYA_0219*	*cpcA* -	CTTTAAAGctcCGTGAGATcgcCTAGATCC	-109	8.70	phycocyanin, alpha subunit
18	*CYA_2357 CYA_2356*	*petM panE*	CTCAACCTctcCTTAAAAAcatGATGAGGA	-474	8.66	cytochrome b6-f complex, subunit VII
19	*CYA_1627*	-	GTTGATTTtaaGTTTAATTgcaATTAAAAA	-700	8.64	peptidase, M23B family
20	*CYA_1593 CYA_1594 CYA_1595*	- - -	ATTAAAAAatgCTTAACTActtGTTGCAAG	-19	8.58	NAD(P)H dehydrogenase, subunit NdhF3 family
21	*CYA_1061*	-	TTTAAGGTtagGTCAAGCGcagGTTAAGCT	-356	8.54	hypothetical protein
22	*CYA_0664*	-	GTTAACCGgagACTTGCTTgttATTTTTGT	-98	8.54	hypothetical protein
23	*CYA_0483*	-	CTTAAGCCttcCATAACCTctgCCTAGGTG	-145	8.50	GGDEF/FHA domain protein
24	*CYA_0702*	-	GTTGAGCAgccCTTCATTTcttCTTGTCCG	-77	8.43	polyA polymerase family protein
25	*CYA_1688 CYA_1689 CYA_1690*	- - *radC*	TTTGATTGcgaATTCAGGGcccCTTCAACG	-213	8.38	hypothetical protein
26	*CYA_2305*	-	GTTAGCCGaggCTTATTCCtggGGTACCGT	-724	8.35	antioxidant, AhpC/TSA family

Table 3s. Predicted Pho boxes for Bprime

Rank	Transcription unit	Names	Pho Boxes	Position	Score	Annotation of the first gene in the transcription unit
1	*CYB_1915 CYB_1914 CYB_1913 CYB_1912 CYB_1911*	*pstS pstC pstA pstB -*	TTTTCTCTtgaCTTAACCCaaaGTTAACCT	-67	10.96	phosphate ABC transporter, phosphate-binding protein
2	*CYB_1988*	-	CTTAATCTgagCTTAGTCTtctCTTAACTT	-135	10.49	hypothetical protein
3	*CYB_0684*	*phoD*	GTTAAACCaacCCTAACCCaacCTTCACCT	-114	10.21	alkaline phosphatase
4	*CYB_0159 CYB_0160 CYB_0161 CYB_0162 CYB_0163*	*phnC phnD phnE - phnH*	CTTAACTTggtTTTAACTTcagTATCAGGC	-58	10.20	phosphonate ABC transporter, ATP-binding protein
	CYB_0164 CYB_0165 CYB_0166 CYB_0167 CYB_0168	*phnI phnJ phnK phnL -*				
5	*CYB_2221 CYB_2220*	- -	CTTAACCTggaCATAACCTtgcCTTTACTT	-34	10.14	hypothetical protein
6	*CYB_0246*	-	TTTAATGCtgtTTTAATATcttCTTAACGT	-120	9.98	4-hydroxyphenylpyruvate dioxygenase, putative
7	*CYB_2465 CYB_2466*	- -	TTTAACTTtttCTTTAACTtttCTTTTGAA	-108	9.98	sensor histidine kinase
8	*CYB_0245*	*pflB*	GTTAAGAAgatATTAAAACagcATTAAAAA	-263	9.95	formate acetyltransferase
9	*CYB_1898*	-	ATTTACCCtacCTTCAGGCtagCTTAATCT	-121	9.50	ABC transporter, ATP-binding protein
10	*CYB_2765 CYB_2766*	*nucH -*	CTTAAGCGtacCTTAACTGcccCTTTACTC	-91	9.49	endonuclease/exonuclease/phosphatase family protein
11	*CYB_2627*	-	GTTACCCGtatCTTAACCCttcTTTACGCT	-140	9.24	hypothetical protein
12	*CYB_1079*	-	GTTAAATCgagGTTAAGCCcagGTTATCCT	-133	9.20	hypothetical protein
13	*CYB_1942 CYB_1941*	- -	TCTCAAACtttGTTTAAGGttaCTTAAACT	-143	9.10	electron transfer flavoprotein, beta subunit/FixA family protein
14	*CYB_2516*	*napA*	TTTGATGTgctTTTAAGTActtATTAAACG	-129	9.04	transporter, monovalent cation:proton antiporter-2 (CPA2) family
15	*CYB_1351*	-	TTTCAACTtttATTACCATctaTTTCAACT	-229	9.01	hypothetical protein
16	*CYB_1412*	-	TTTAAGCCaagATTGACTGagtCTTTATTC	-250	8.96	GDSL-like lipase/acylhydrolase domain protein
17	*CYB_1486*	-	GTTGGTATcttTTTTAGATtatCTTAGAAG	-98	8.84	putative small GTP-binding protein
18	*CYB_1198 CYB_1199*	- -	GTTAAGAGataGTTAAGAGataGTTAACAA	-159	8.84	alkaline phosphatase
19	*CYB_2597 CYB_2598 CYB_2599 CYB_2600 CYB_2601*	*rplC rplD rplW rplB rpsS*	CTTTTCTTccaCTTGAGCTgccCTTACCCT	-454	8.79	ribosomal protein L3
	CYB_2602 CYB_2603 CYB_2604 CYB_2605 CYB_2606	*rplV rpsC rplP rpmC rpsQ*				
	CYB_2607 CYB_2608 CYB_2609 CYB_2610 CYB_2611	*rplN rplX rplE rpsH rplF*				
	CYB_2612 CYB_2613 CYB_2614	*rplR rpsE rplO*				
20	*CYB_2662 CYB_2663*	- -	TTTCATCTcagCTTAACTTtgtCAAGAAAT	-173	8.79	ISSoc13, transposase orfA
21	*CYB_1325*	-	TTTTATATcgaCTTAAGATagcATTATCGG	-137	8.75	hypothetical protein
22	*CYB_0689 CYB_0690*	- -	GTTACCCTccaATTAATCTccaATTAATTC	-150	8.68	hypothetical protein
23	*CYB_1747 CYB_1749*	*- proC*	GTTAAATTgtgATTAAATTttgTGTAAAGC	-542	8.53	hypothetical protein
24	*CYB_0102 CYB_0101 CYB_0099*	- - -	CCTCAGCTcagGTTAACCGaccCTTAGATT	-318	8.53	beta-carotene ketolase, putative
25	*CYB_2515*	-	TTTAATAAgtaCTTAAAAGcacATCAAATA	-114	8.52	serine/threonine protein kinase
26	*CYB_0572*	-	TTTATCGAatgCTTAGCCAttcATTTTGTT	-112	8.50	putative bacteriochlorophyll 4-vinyl reductase
27	*CYB_1147*	-	TATGAACTtaaGTTAACATtgcCTTCCCCT	-40	8.43	glycosyl hydrolase, family 57
28	*CYB_2032*	-	CTTCTCTCcaaATTAACGGaggCTTATCCA	-165	8.42	hypothetical protein
29	*CYB_2628 CYB_2629*	*msrB -*	CGTAAAGAaggGTTAAGATacgGGTAACCT	-93	8.36	methionine-R-sulfoxide reductase

Table 4s. Predicted Pho boxes for BF-1

Rank	Transcription unit	Names	Pho boxes	Position	Score	Annotation of the first gene in the transcription unit
1	*tlr0786*	-	ATTAACCTaggATTAACCTattTTTAACCC	-228	10.52	hypothetical protein
2	*tlr2164 tlr2165 tlr2166 tlr2167*	*sphX pstC pstA pstB*	TTTAAACAaacTTTTACCTtctCTTAACTT	-23	9.91	periplasmic phosphate-binding protein of phosphate ABC transporter
3	*tlr0711*	-	CTTTGCCCcgcCTTAACAGaatCTTAAACT	-151	9.36	D-lactate dehydrogenase
4	*tlr0862*	-	ATTAATCTattCTTAAACCaaaTTTAATTC	-123	9.16	hypothetical protein
5	*tsl0785*	-	GTTAAGCTaggGTTAAAAAtagGTTAATCC	-35	8.95	hypothetical protein
6	*tsr1443 tlr1444 tlr1445 tlr1446 tlr1447*	- - *ycf63* - -	GATAGCAGtggATTAATTTcctTTTAACCT	-130	8.94	RNA binding protein
	tlr1448 tlr1449	*feoB cobI*				
7	*tlr1653*	*desC2*	ATTAAGAAagcGTTTACAGctgCTTCTGCC	-49	8.85	acyl-CoA desaturase 3
8	*tll0860*	-	ATTAAATTtggTTTAAGAAtagATTAATTT	-669	8.81	hypothetical protein
9	*tlr0241 tlr0242 tlr0243*	- - -	GTTTATCGtgcCTTTAAATttaTTTACCTT	-167	8.80	hypothetical protein
10	*tsl1609 tll1608*	- -	TTTAATGActgAATTAACTtgaATTAACTT	-114	8.74	hypothetical protein
11	*tll1774*	-	GTTAAATAatcCTTAAATTcttTTTGATGG	-384	8.72	N-acetylmuramoyl-L-alanine amidase
12	*tlr1157 tlr1158*	- -	TTTAATGCagtCATAAACTtttGCTAAAGC	-133	8.71	hypothetical protein
13	*tll2359*	*fus*	GTTAACCCtaaCTTAACGGttgTTTACAGC	-149	8.66	elongation factor EF-G
14	*tlr0658 tlr0659*	- -	GTTGACTCacaATTAAACCcgtCTTAGACA	-110	8.64	enolase
15	*tlr2169 tlr2170 tlr2171*	- - -	TTTTTGCAaatCTTGATCGaaaCTTAAACA	-21	8.51	cyanophycinase
16	*tlr0027 tlr0028*	- -	CTTAACATcagTTTTGCCTataGTTATAGC	-33	8.46	hypothetical protein
17	*tlr1930 tlr1931*	- -	GTTTACATttcGTTACATTactCTTCAGTT	-29	8.29	putative glycosyl transferase
18	*tsr0373 tlr0374 tsr0375*	- - -	CTGAAGCAagcCTTAATAGaacCTTAATTT	-426	8.28	hypothetical protein
19	*tlr1853 tlr1854 tlr1855 tlr1856*	- - - -	GTTGTTGCctcTTTAGCTTggcCTTGATTG	-24	8.18	moaA/nifB/pqqE family protein
20	*tll1025 tll1024 tll1023 tll1022 tll1021*	- - - - -	ATTTATAGactTTTAAAGCggcGGTTGTCT	-56	8.16	two-component response regulator
21	*tlr2085*	-	ATTAATTGttaCATCAGCAtcaTTTCAAGG	-55	8.15	hypothetical protein
22	*tll0141*	-	TTTTATAGtcaGTTGTCAAttaATTGTCCT	-228	8.12	hypothetical protein
23	*tlr2360*	-	TGTAAACAaccGTTAAGTTaggGTTAACGT	-24	8.11	hypothetical protein
24	*tlr1669*	-	TGTGAAAAtaaCTTTATTAagaTTTATTAA	-216	8.10	hypothetical protein

Table 5s. Predicted Pho boxes for IMS101

Rank	Transcription unit	Names	Pho boxes	Position	Score	Annotation of the first gene in the transcription unit
1	*Tery_3845*	-	TTTAACCAaagCTTAAACGaaaTTTAAACC	-173	10.12	protein of unknown function DUF839
2	*Tery_0505 Tery_0506 Tery_0507 Tery_0508*	- - - -	CTTAAAATaatCGTAAATTaagGTTAAAAT	-19	9.35	protein of unknown function DUF124
3	*Tery_2377 Tery_2378*	- -	ATTTACCAacgCTTAATTTtttCTTAACCT	-191	9.17	efflux transporter, RND family, MFP subunit
4	*Tery_0988 Tery_0987*	- -	ATTAACTAaacATTCAACTttaATTGACTT	-330	8.89	Ankyrin
5	*Tery_2737*	-	TTTAATGAtcaATTAATCAtgcTTTAATCC	-36	8.85	hypothetical protein
6	*Tery_5022*	-	CTTACTACataATTAACTTtaCTTAAACA	-510	8.65	Carboxymuconolactone decarboxylase
7	*Tery_4986 Tery_4987*	- -	ATTTACTCtaaTTTCAGTTtttCTTATTCT	-503	8.65	TRAP transporter solute receptor, TAXI family
8	*Tery_3566 Tery_3567 Tery_3568*	- - -	ATTAAAAAtatCTTTAGCTtatATTACTAT	-278	8.61	hypothetical protein
9	*Tery_3016*	-	ATTTATCTtaaTTTAACCTagtTTTACTAA	-681	8.52	Peptidoglycan glycosyltransferase
10	*Tery_3754 Tery_3753*	- -	TTTATAAGtgcCTTAATCTttgCCTTAACT	-19	8.38	methyltransferase FkbM family
11	*Tery_3993*	-	CTTTATTTttaCTTATACTtatATTATAAA	-82	8.37	adenylate/guanylate cyclase
12	*Tery_1101*	-	TTTTATGAtaaCTTAATTGataTTTAAAGT	-49	8.36	Insertion element protein
13	*Tery_3826*	-	ATTAAATAactCTTAAACCaaaATTTGAAA	-27	8.33	Dimethylaniline monooxygenase (N-oxide forming)
14	*Tery_0813*	-	ACTATCCAttgTTTGACTTattCTTGATAA	-118	8.33	hypothetical protein
15	*Tery_0406*	-	GTTAAGTTtagCTTGAATTtatCTTGACAT	-749	8.29	formate dehydrogenase family accessory protein FdhD
16	*Tery_1035*	-	ATTTTCTGaaaTTTAACTAcaaCTTAGAAT	-68	8.26	hypothetical protein
17	*Tery_2828 Tery_2829*	- -	TTTAACAGtaaTTTTAGCTcacATTTAAAA	-401	8.26	Uncharacterized protein with protein kinase and helix-hairpin-helix DNA-binding domains-like
18	*Tery_2902*	*sphR*	GATAACCCaaaGTTAACCTtttCTTAACCT	-337	8.26	two component transcriptional regulator, winged helix family
19	*Tery_3048*	-	TTTAACAAaatATTAACCTaggAATTAATA	-247	8.21	Cna B-type
20	*Tery_1874*	-	GTTTGAATttcATTAAGCAattAATATCCA	-277	8.20	cell surface protein
21	*Tery_3534*	*sphx*	TTTGATATtttTTTAACCTgttCTTAATCT	-164	8.18	phosphate ABC transporter, periplasmic phosphate-binding protein
22	*Tery_2317*	-	TTTGACGAtgcCATAAATTtttGTTAATTT	-186	8.17	hypothetical protein
23	*Tery_2109*	-	ATTAAATAataTTTTACCTattATTACTAA	-471	8.15	AMP-dependent synthetase and ligase
24	*Tery_3699*	-	GTTAAGGAaagCTTAAAGTattTTTAAGCT	-80	8.11	amidophosphoribosyltransferase
25	*Tery_3585 Tery_3584 Tery_3583 Tery_3582*	- - - -	GTTATAAAacaTTTAAAAGtaaCTTATTTG	-141	8.11	GCN5-related N-acetyltransferase
26	*Tery_4993 Tery_4994 Tery_4995 Tery_4996*	*phnD phnC phnE phnE2*	GAAAACAAgttTTTAACCTcttCTTAAACT	-435	8.09	phosphonate ABC transporter, periplasmic phosphonate-binding protein
	Tery_4997 Tery_4998 Tery_4999 Tery_5000	*phnG phnH phnI phnJ*				
	Tery_5001 Tery_5002 Tery_5003	*phnK phnL phnM*				
27	*Tery_0622*	-	TTTAACATaaaTTTTTACTtttTTTTAGAT	-419	8.08	hypothetical protein
28	*Tery_2104*	-	CTTAAACTtttTTTGGTCAatcCTTACTCT	-687	8.07	hypothetical protein
29	*Tery_4515 Tery_4514*	- -	GTTAATCAttaCTTTTAAAgctCTTAACTT	-655	8.04	Carbohydrate-selective porin OprB
30	*Tery_2219*	-	CTTCAATAactATTAAACTcctCTTGAAAT	-230	8.04	hypothetical protein
31	*Tery_3877*	-	TTTAAACTattTTTAAACTattTTTAAGAT	-600	8.03	exonuclease SbcC
32	*Tery_0420*	-	ATTATCAGggcTTTAACCAtttTTTTCCCT	-651	8.01	Glyoxalase/bleomycin resistance protein/dioxygenase
33	*Tery_2914*	-	TTTATTCTatgATTAATCAagtCTTCACCA	-177	8.00	short-chain dehydrogenase/reductase SDR
34	*Tery_4360*	-	TTTAATTAattCTTAATTTtttTTTTACTG	-465	7.99	hypothetical protein
35	*Tery_3324*	-	TTTTTTACttcCCTAATCTcatCTTGCTTT	-37	7.94	hypothetical protein
36	*Tery_2389 Tery_2388*	- -	GTTAACCTaaaCCTTATGTtaaGTTACCTA	-52	7.94	hypothetical protein
37	*Tery_4221 Tery_4222*	- -	ATTAATTTttcTTTAAAAAgagCTTTTTCT	-283	7.94	PAS/PAC sensor signal transduction histidine kinase
38	*Tery_4676 Tery_4677*	- -	TTTTAAATataCTTAACCTaaaATTAATAT	-330	7.93	hypothetical protein
39	*Tery_2787*	-	ATTAACCTactTTTAACCCaagATTGAGGT	-175	7.91	Ferritin and Dps
40	*Tery_0345*	-	GTTAAATAttgCTTAAATAgatATTCATTA	-612	7.89	hypothetical protein
41	*Tery_1792*	-	TTTGATTTggaCTTATACTtggACTTACTC	-127	7.89	3'(2'),5'-bisphosphate nucleotidase
42	*Tery_2555*	-	ATTAGTAAtatATTAAATGagtCTTTTGTT	-486	7.88	hypothetical protein

Table 6s. Predicted Pho boxes for PCC6301

Rank	Transcription unit	Names	Pho boxes	Position	Score	Annotation of the first gene in the transcription unit
1	*syc0545_d*	*nucH*	TTTAAAGTgctGTTAATCCttcCTTTACCG	-276	9.76	hypothetical protein
2	*syc1661_d syc1662_d syc1663_d syc1664_d*	*sphX pstS pstC pstA*	CTTAGGGTcgcCTTAATAGgctGTTAAACT	-48	9.75	periplasmic phosphate-binding protein of ABC transporter
	syc1665_d syc1666_d	*pstB pnp*				
3	*syc0699_c*	-	GTTTAACCtgaCTTAATTCtggATTGAATA	-73	9.49	hypothetical protein
4	*syc0163_d*	*phoA*	TTTAACTAtttCATAATCTattCTCAATCT	-216	9.41	alkaline phosphatase
5	*syc1524_c*	-	GTTGATTTggcTTTAGTCTcctATTGATAT	-21	9.25	hypothetical protein
6	*syc2253_d syc2254_d*	*murF* -	ATTAACATagtCTTATCCTggcATTAGTCA	-90	9.18	UDP-N-acetylmuramoylalanyl-D-glutamyl-2 6-diaminopimelate--D-alanyl-D-alanine ligase
7	*syc0685_c syc0684_c syc0683_c*	*pixG* - -	TTTAACTTggcCTTAATCTtcgAATAGGTA	-85	9.08	two-component response regulator rre36 homolog
8	*syc1209_c*	-	TTTAAGCCcagCTTTAGCTcagCCTGGGAC	-84	8.95	probable potassium channel protein
9	*syc1561_c*	-	TTTCTGCGgtcCTTAAACGagcCTTCTAGT	-34	8.93	hypothetical protein
10	*syc2437_c*	-	TTTGACAGcttTTTATCTAagaCTTATTCT	-32	8.80	rubrerythrin
11	*syc1620_d*	-	CTTCATTGagaCTTGACCTagcGCTTTGGT	-122	8.68	hypothetical protein
12	*syc1747_d*	*cysR*	ATAGAAAAggaATTAACCAaacCTTAAAGT	-71	8.64	transcriptional regulator CRP family protein
13	*syc0964_c*	-	TTTCACTGagcCTTAACATtccCTTCGCGA	-393	8.54	hypothetical protein
14	*syc2439_c*	-	GCTCAACAtctTTTCAATGattCCTGAATA	-94	8.51	hypothetical protein
15	*syc2138_d*	-	CTTAACCGcttCTGAATCTaagCCTGCTAT	-30	8.50	hypothetical protein
16	*syc1338_c*	*glnN*	TCTAATTTtctCTTCAAAAggaATTTAAAT	-195	8.47	glutamine synthetase type III
17	*syc1093_c*	*psbAI*	CTTAGCTAaaaATTAAGGGtttTTTACACC	-79	8.35	photosystem II D1 protein
18	*syc1447_d syc1448_d*	- -	GTTGGCACaaaATTCATCTtttACTAATCT	-127	8.34	hypothetical protein
19	*syc1445_d*	-	TTTAAAAAataCTTTCTAActcAATTCACA	-513	8.26	hypothetical protein
20	*syc0023_d syc0024_d*	- *ppk*	TTTTTGCCcatTTTAAATAactTTTGCTTT	-583	8.21	hypothetical protein
21	*syc2044_d syc2045_d*	*psaA psaB*	CCTGAAAGctcTTTGAATTattGTTAATCG	-277	8.20	photosystem I P700 chlorophyll a apoprotein subunit Ia
22	*syc1030_d*	-	TTTAAGGAcaaGATATGCTggtTTTTACCA	-307	8.15	aldehyde dehydrogenase
23	*syc1953_c*	-	TTTATCATtttATTGAGCTtcaCTTACAGA	-511	8.07	probable glutathione S-transferase
24	*syc1940_d syc1941_d*	- -	ATTTAAGAatgCTTGTTCTtaaAGTTGAAA	-21	8.05	hypothetical protein
25	*syc0266_c*	-	CATGGCTCgagTTTTAGCTcttCATAACCT	-170	8.05	similar to S-layer-RTX protein
26	*syc0197_d syc0198_d syc0199_d syc0200_d*	- *phy* - -	GTTAAACTcccCCTAAGCAcctTCTAGCTC	-45	8.02	hypothetical protein
27	*syc0467_c*	-	TTTGAAAAattTTTATGGCtggATTAATCT	-415	8.01	cation-transporting ATPase PacL homolog
28	*syc0006_c*	-	CTTAACAAataATTAACTCccgTATTTTCG	-33	7.99	probable amidase
29	*syc2462_d*	-	TTTTATCAatgATTAATGAttgCTTTGGGA	-376	7.96	hypothetical protein
30	*syc2384_c*	-	TTTTACCAaatTTTGAGCTcagTTTTCGCG	-113	7.96	cell division protein Ftn6 hypothetical protein
31	*syc1254_c*	-	CTTGATAGctcTTTAGTCTagaTGTAAACA	-108	7.95	hypothetical protein
32	*syc0087_d*	-	CTTAAATCcttTTTAGTGTcaaATTTTCTC	-247	7.92	hypothetical protein
33	*syc1997_c*	-	TGTTTACTttcATTGAAATaaaTTTCAGCT	-192	7.90	hypothetical protein

Table 7s. Predicted Pho boxes for PCC6803

Rank	Transcription unit	Names	PhoB boxes	Position	Score	Annotation of the first gene in the transcription unit
1	*sll0679*	*sphX*	TTTAACCAaacCTTTACTAgggCTTAACCT	-105	10.31	SphX protein precursor
2	*slr1247 slr1248 slr1249 slr1250*	*pstS2 pstC2 pstA2 pstB2*	CTTAATTCtatCTTAATTTcgaCTTAATCA	-303	10.1	periplasmic phosphate binding protein
3	*sll1552 sll0720*	- -	CTTAATTTtaaTTTAACCTtaaTTTAATCT	-698	9.817	hypothetical protein
4	*sll1555*	-	CTTAAATTttcCGTAATCTcttCTTAAAGT	-246	9.181	hybrid sensory kinase
5	*slr0753*	*p*	CTTAATCAcggCTTAGTTTaatTTTAATCT	-25	8.96	P protein
6	*sll1202 sll1203 sll1204*	- - -	ATTAAAAGttaCTTGCTGTtttTTTAATGA	-99	8.814	hypothetical protein
7	*sll0654 sll0656*	*phoA nucH*	CTTAACCTtttCATAGTCTaacCATAAGTT	-156	8.801	alkaline phosphatase
8	*slr1908*	-	GTTAAGTAaagGTTAAGGAtccGTTAAGCA	-339	8.745	hypothetical protein
9	*slr2110 slr2111 slr2112 slr2113 slr2114*	- - - - *spsC*	TTTAAAGGgctTTTTTTCTcttTATAACTA	-137	8.681	hypothetical protein
	slr2115 slr2116 slr2117	- *spsA* -				
10	*slr0793 slr0794 slr0795 slr0796*	- - - *nreB*	TTTCACCTgaaTTTCATACcccCTTTGGCA	-42	8.649	hypothetical protein
11	*slr0907 slr0909 slr0912 slr0913 slr0914*	- - - - -	CTTGCACAgctTTTAACCAcagCTTAAGAG	-335	8.634	hypothetical protein
12	*sll0741 sll0742 sll0743 sll0744 sll0745*	*nifJ* - - - *pfkA*	TTTAAACTataGTTAATTTggcTGTAAATT	-524	8.525	pyruvate oxidoreductase
13	*ssr2333 slr1392*	- *feoB*	CTTTTCCAtagGTTGCATGgaaTTTAATCA	-108	8.469	hypothetical protein
14	*sll0680 sll0681 sll0682 sll0683 sll0684*	*pstS1 pstC1 pstA1 pstB1 pstB1'*	ATTCCATAgacCTTAACCTtccCTTTACCA	-73	8.464	phosphate-binding periplasmic protein precursor
	sll0685	-				
15	*slr0449*	- -	TTTTTCCTtgaCTTAAGTCaaaAATAACCT	-283	8.447	DNR protein
16	*slr1114 slr1115 slr1116 slr1117*	- - - -	ATTTATTTtccATTTATCTgagATTTATTT	-190	8.398	hypothetical protein
17	*sll0676 ssl1277 sll0677 sll0678*	- - - -	TTTAAAAGgtaATTTTTTAaaaACTAAACT	-333	8.395	hypothetical protein
18	*sll1876*	*hemN*	GTTTATTTcaaTTTAATACtctCTTTTGCA	-206	8.311	oxygen independent coprophorphyrinogen III oxidase
19	*slr1670 slr1672 slr1673 slr1674*	- *glpK spoU* -	GTTAAAGAttaCTTAACCCgtcAGTAAAAT	-535	8.269	hypothetical protein
20	*slr1651*	*hlyB*	ATTAATATaagATTAAATTaagGTTAAATT	-100	8.244	haemolysin secretion ATP-binding protein
21	*sll1491*	-	TATAAAAAgacTTTAAGTAacgCTTATTAT	-43	8.239	beta transducin-like protein
22	*sll1169*	-	TGTCAATAtttTTTCACTAaatATTTATTT	-597	8.236	hypothetical protein
23	*slr1068 slr1069 slr1070 slr1071 slr1072*	- - - - *yefA* -	TATAGAAGttaTTTATCCAccgATTAACCA	-237	8.21	hypothetical protein
	slr1074 slr1075	- -				
24	*sll1849*	-	GTTAAAAGtaaCTTCTTAGcagAGTGATAA	-60	8.207	hypothetical protein
25	*slr0222*	-	ATTTTCCTtgtCTTGATCAatgTTTGACCT	-10	8.175	hybrid sensory kinase
26	*slr1181*	*psbA1*	ATTAATCTgaaATTCAAGGgaaGTTAATCA	-141	8.171	photosystem II D1 protein
27	*sll1205*	*pchR*	ATTGATCAattCTTTCAATctaCTTCAGCT	-32	8.159	regulatory protein; PchR
28	*ssr1562*	-	GTTGGCAAaacCTTAACAAaaaGTTTAGAG	-128	8.137	hypothetical protein
29	*sll1525*	*prk*	CTTGACAGgggGTTAACAAgccCATAGGCT	-91	8.122	phosphoribulokinase
30	*slr1712*	-	TTCAACCAttaCTTTGACTgaaTTTGATGC	-116	8.113	hypothetical protein

Table 8s. Predicted Pho boxes for PCC7120

Rank	Transcription unit	Names	PhoB boxes	Position	Score	Annotation of the first gene in the transcription unit
1	*alr0132*	-	ATTAAAGTaatCTTTAACTagtCTTAACCT	-330	9.96	hypothetical protein
2	*alr1170 alr1171*	- -	GTTAATTAcctCTTAAATCcggTTTAATCT	-327	9.68	two-component response regulator
3	*alr1386*	-	AGTAACCTgttCTTACCTTgctCTTAACTA	-56	9.47	hypothetical protein
4	*all4382*	-	TTTAACTTgttATTATCCTtccTTTAATCA	-169	9.32	hypothetical protein
5	*alr5197 alr5198*	- -	CTTAACCTtgaATTAATGTtagTTTAACCC	-286	9.31	hypothetical protein
6	*alr5291*	*phoA*	ATTAATCTttaGTTAACCTgaaTTTATATT	-223	9.25	hypothetical protein
7	*all2230 all2229 all2228 all2227*	*phnC - phnD phnE*	TTTAGTATtttCTTAATTTtttATTAACCG	-45	9.14	ABC tranporter, ATP-binding component
8	*all0542*	-	ATTATGATcaaGTTATAAAaacTTTAAACT	-234	9.13	two-component sensor histidine kinase
9	*alr4693*	-	TTTGTACGaacTTTAACTCattCTTTGCTT	-309	9.00	hypothetical protein
10	*all4575 all4574 all4573 all4572*	*pstS1 pstC1 pstA1 pstB1*	TTTTCTCTtgaTTTAACCTttcGTTAACCT	-64	8.97	phosphate-binding periplasmic protein of phosphate ABC transporter
11	*all5196 all5195 all5194 all5193 all5192*	- - - - -	GTTAAACTaacATTAATTCaagGTTAAGTA	-364	8.97	hypothetical protein
12	*alr4976*	*phoD1*	CTTAACATcgtCTTAAACCctaCTTGATGA	-86	8.89	phosphodiesterase/alkaline phosphatase D
13	*alr3466*	-	ATTTACAAcagTTTCAGCTtatTTTTACAA	-407	8.83	WD-40 repeat protein
14	*all4085*	-	ATTAAGCAataATTTATTTattCTTAAGAT	-111	8.81	endonuclease V
15	*all0471 all0470 all0469 all0468*	- - - -	TTTAACATaatGTTAAGATtgcATTAACTC	-158	8.80	hypothetical protein
16	*alr3397*	-	ATTTTACTattATTAATCTctaGATAATCT	-364	8.80	phosphoenolpyruvate synthase
17	*alr1546*	-	GATAAATAataATTAATCTtttCTTTATCT	-658	8.75	hypothetical protein
18	*all2006*	-	TTTAATGTataCTTAACCTattGTTAACCT	-38	8.74	hypothetical protein
19	*all0274*	-	TTTAATCTtttGATAATCTtttGATAAATA	-116	8.72	hypothetical protein
20	*all1750*	-	GTTAAAAAgatATTTACTAaccCCTAACCT	-41	8.71	hypothetical protein
21	*alr3874*	-	GTTAAGGCttaTTTAAAGTtcgGTTAATTA	-44	8.68	hypothetical protein
22	*alr4794*	-	ATTAATCAataTATAACCTgacGTTAACAC	-239	8.62	hypothetical protein
23	*alr4823*	-	CTTAATTAaaaTATAACCAaaaCTTAAAAT	-144	8.61	hypothetical protein
24	*all4220*	-	ACTTAAAAataTTTAACAGtaaATTAATCC	-191	8.50	hypothetical protein
25	*all0638 all0637*	- -	CTTTAAATaacGTTTATAAttgCTTTACCT	-279	8.45	two-component hybrid sensor and regulator
26	*alr0851*	-	CTTTAGCTtccCGTAGGGTattTTTAATTA	-17	8.44	lysophospholipase
27	*asl0826 all0825 all0824 all0823 asl0822*	- - - - -	CCTAAACCcttATTTAAAGcagCTTAACTT	-7	8.38	hypothetical protein
28	*all3564*	-	CTTTATCTtgaCTTATCTAtaaATTATAAA	-363	8.35	hypothetical protein
29	*alr0208*	-	TTTCACCTtaaATTAAAGTttaCTTAACCT	-172	8.34	hypothetical protein
30	*alr2234*	*phoD*	ATTAACCTtatCTTAGTCAacaATTAATTT	-258	8.34	phosphodiesterase/alkaline phosphatase D
31	*alr0430*	-	ATTAACAAattATTACCATtagATTGGATT	-406	8.32	hypothetical protein
32	*alr2233*	-	TTTTACCTttcATTAAATTttaCTTAAACT	-195	8.31	hypothetical protein
33	*all1844*	-	TATTAAAAttgCTTAACACttcGTTACACT	-11	8.29	hypothetical protein
34	*all2704 all2703*	- -	TTTAAAGTctgTTTACTCAcaaTTGAAATT	-59	8.28	hypothetical protein
35	*alr1113 alr1114*	- -	ATTTAATTaacTTTTAACTacaTTTAATAA	-156	8.28	ParA family protein
36	*all5069*	-	TTTAAATAgagCTTAAAGCaacATTGAAGT	-128	8.27	two-component response regulator
37	*alr5224*	-	TTTATTTCaatATTAATAAgttCTTTATCT	-172	8.27	hypothetical protein
38	*alr1094 alr1095*	*sphX gap3*	CTTTACTAataCATCAACTtttATTGATGA	-106	8.19	periplasmic phosphate-binding protein of ABC transporter
39	*all3132 all3131*	- -	ATTAAACAaaaGTTCTCCAtagTTTGCCAA	-316	8.18	ABC transport protein, ATP-binding subunit
40	*alr1677*	-	ATTAACTGttaATTAAACCgtgGGTAAAAG	-216	8.17	hypothetical protein
41	*all2550 all2549*	- -	CTTGAAAAaatCATCAACAaaaATTAGCAA	-82	8.17	hypothetical protein
42	*all0275*	-	GTTCTTGTgaaCTTATTTTtttCTTAATCT	-122	8.17	glycerophosphoryl diester phosphodiesterase
43	*asr2932 alr2933*	- -	GTTAACAAtagGTTGAACAtatTTTAAGGA	-23	8.13	hypothetical protein
44	*alr1997 alr1998*	- -	GTTTCTGAgagTTTTACTGctaGCTAACCT	-227	8.11	hypothetical protein
45	*all1082*	-	TTTAAAGTtgtCTTGACTGacaCATAATCA	-288	8.11	hypothetical protein
46	*alr0169*	-	TTCTAGCAaagCTTAATCAcaaCTTACCCG	-126	8.11	cyclomaltodextrin glucanotransferase
47	*all1239*	-	TTTTACGCcatGATACTCTgatTTTTACTA	-510	8.11	hypothetical protein
48	*all4118*	-	TTTATCTGaatATTAAAGCtatCTTAAGCC	-313	8.11	hypothetical protein
49	*all3873*	-	ATTAACCGaacTTTAAATAagcCTTAACTG	-212	8.09	hypothetical protein
50	*asl0795 all0794*	- -	CTTTAGCGgggGTTTGTCTtgcCTTATTCC	-132	8.07	hypothetical protein
51	*alr0276*	*nucH*	TTTTACCTtctCCTATTATtctCTTAATCT	-178	8.05	hypothetical protein
52	*alr0528 alr0529 alr0530 asr0531 alr0532*	*cpcB cpcA cpcC cpcD cpc*	TTTTTGCAactATTAAAAAataATTAAGAC	-412	8.02	phycocyanin beta chain
	alr0533 alr0534 alr0535 alr0536 alr0537	*cpcF cpcG1 cpcG2 cpcG3 cpcG4*				
53	*alr5272*	-	CTTAATTAaatCTTAATAAtaaGTTTCAGT	-238	8.00	two-component sensor histidine kinase
54	*all1996 all1995 asl1994 asl1993*	- - - -	AGTTATTGtttCTTAATTTtgcATTGGTCT	-131	7.99	hypothetical protein
55	*alr4525 alr4526 asr4527 alr4528 alr4529*	- - - - - - - - - - -	TTTATGTAtttCTTAAATAttaATTAAAGC	-107	7.98	hypothetical protein
56	*all2509*	-	ATTCTCTAttcCTTAAGCCtgaTTTTTTCG	-601	7.98	hypothetical protein
57	*alr3823 alr3824*	*radA -*	TTTGAGCAataCTTAAGGAaaaATTAAGAT	-38	7.97	DNA repair protein

Table 9s. Predicted Pho boxes for PCC7421

Rank	Transcription unit	Names	PhoB boxes	Position	Score	Annotation of the first gene in the transcription unit
1	*glr0275*	-	GTTAATCGcagGTTGACCTgctCTTAATCT	-69	11.79	hypothetical protein
2	*gll0490*	*phoD*	CTTAACCAggaAATAAAGGggcATTAACGG	-68	11.23	phosphodiesterase/alkaline phosphatase D
3	*glr4430*	-	TTTTCACTatcATTAATCTtgtGTTAACCC	-207	10.40	hypothetical protein
4	*gll0367 gll0366 gll0365*	- - -	GTTTATCCtgaCGTAAACAtagCTTCAACT	-76	9.92	aquaporin Z
5	*gll0893*	*phoA*	TTTAACTTtgaCTTAGTGGtttCATAATCT	-146	9.89	hypothetical protein
6	*gll3960*	-	CTTAACCGggcCTTCAACTgctCCTTACCT	-34	9.84	hypothetical protein
7	*glr2446*	-	ATTAATGCggcCTTAACGGgggATTATCCG	-72	9.83	hypothetical protein
8	*gll4200*	-	CTTAAACCaatAATAATATtgaATTTTTTC	-186	9.69	similar to cell surface glycoprotein (s-layer protein) related protein
9	*glr0280 glr0281 glr0282*	- - -	GTTAACCAgtgCTTATTCAtccGTTACGGG	-81	9.61	hypothetical protein
10	*glr4163 glr4164 glr4165 gvip549 gvip550*	- - - *secD secF*	GTTGTCTTatgTTTGAATCtttCTTAACTT	-140	9.57	hypothetical protein
11	*glr0453 glr0454 glr0455 glr0456 glr0457*	- - - - -	TTTAAACAcgaCATAGAGAaaaCCTAACAT	-714	9.52	hypothetical protein
	glr0458 glr0459	- -				
12	*gll3250*	-	GTTTAGAAtacCTTAAGTCaccACTGAGCC	-631	9.37	hypothetical protein
13	*gsr2660*	-	ATTAAGACctgCTTAACTTaatAATGTGTA	-40	9.33	hypothetical protein
14	*glr0997*	-	CTTAACTAtccTTTAACTGggtTTTGAGAC	-53	9.12	hypothetical protein
15	*gll1281 gsl1280*	- -	CTTGGCCGgttATCAATCTcatGCTAACCT	-39	9.07	hypothetical protein
16	*glr3201*	-	GTTAACAAtatGTTCACCGcctGTTAATGC	-118	9.06	hypothetical protein
17	*glr4413*	-	TTTAAACGaaaGTTAACTTcttTTTACCAT	-143	9.06	hypothetical protein
18	*glr0602*	-	CTTAAGGTtggCTTATGCTgcgTGAACTGT	-82	8.97	hypothetical protein
19	*gll2676 gll2675*	- -	GTTAAGTTgacAATGCCGCtcaTTTTACCC	-89	8.93	putative transposase
20	*gll3548*	-	GTTAGCTGattGTTTACTTcgcCATAACCT	-71	8.91	ribulose-phosphate 3-epimerase
21	*gll2960*	-	GATTCATGcatATTAACGCttaGGTAATCC	-642	8.90	hypothetical protein
22	*gll2177*	-	TTTAAGGActcGTCAATCTgagCTTTGTTT	-40	8.90	hypothetical protein
23	*glr2814*	-	TGCGATTGcgaATTAAGGTatcCTTAAATT	-30	8.88	hypothetical protein
24	*glr1552*	-	CTTTCGCGagcATTAATTCagtGTTATCCT	-195	8.88	serine/threonine kinase
25	*gvip107 gvip108*	*rub ycf48*	AGTGAAGAgaaATTAAGTTtttCTTCATAT	-30	8.87	rubredoxin
26	*gll3053*	-	TTTGATGAaatCATGGACAagcGTTCATGT	-33	8.79	hypothetical protein
27	*glr4269*	-	TTTGACGGtaaTTTAATATttcATAGAGGT	-19	8.78	hypothetical protein
28	*gvip499*	*psbO*	ATTTGTCCattTTTCATTGcccTTTGCGCC	-34	8.77	similar to photosystem II manganese-stabilizing polypeptide
29	*glr3061*	-	CTTAAACGgcaTTTTATCGaaaTTTATCCA	-114	8.72	ammonium transporter
30	*gvip126 glr0944*	*hemG* -	TTTCGACCtcaCTTTATGCgcgATTATTCT	-108	8.65	protoporphyrinogen oxidase
31	*glr0445 glr0446 glr0447 glr0448*	*pstS pstC pstA pstB*	GTTAACCTtgcCTTAATCGaatTTCGTTAC	-80	8.64	phosphate ABC transporter periplasmic phosphate-binding protein
32	*glr2842 gsr2843 glr2844*	- - -	ATTTATTTaccGTTTAGACctcGATGGTCT	-139	8.55	hypothetical protein
33	*gll2550 gll2549*	- -	ATTCATACttaCTTGGAATataTGTTATGG	-296	8.54	3-oxoacyl-(acyl-carrier-protein) reductase
34	*gll0074*	-	CTTTACCCattCCTACTGCgccCTTAATCA	-89	8.53	hypothetical protein
35	*glr2447*	-	CTTAATGTcgcGATAGCCTtggGTTTACAC	-72	8.51	hypothetical protein
36	*gsr0742 glr0743*	- -	TTTAGGTTtatTTTGATTCacaGTTAGGCC	-100	8.51	CAB/ELIP/HLIP superfamily protein
37	*gll2466 gll2465*	- -	GTTAGTGCttcTTTAGACCgcaATTCAAAA	-421	8.50	probable glycosyltransferase
38	*gll3924 gvip531 gvip530 gvip529 gvip528*	- *proA rpl22 rps3 rpl16*	TTTCGGTTaaaGTCAACGAagaCTTAATAT	-147	8.48	hypothetical protein
	gvip527 gvip526 gvip525 gvip524 gvip523	*rpl29 rps17 rpl14 rpl24 rpl5*				
	gvip522 gvip521 gvip520 gvip519 gvip518	*rps14 rps8 rpl6 rpl18 rps5*				
	gvip517	*rpl15*				
39	*gsl2445*	-	GATAATCCcccGTTAAGGCcgcATTAATTA	-429	8.47	hypothetical protein
40	*gll4412*	-	GGTAAAAAgaaGTTAACTTtcgTTTAAAGC	-9	8.45	hypothetical protein
41	*glr1387 glr1388 glr1389*	- - -	GTTTTATGgcaATTCACTAagtGTTTACTA	-120	8.41	hypothetical protein
42	*glr3251*	-	TTTTACACaaaTTTATTCTagaGCGAATGG	-23	8.41	hypothetical protein
43	*glr1205*	-	ATTTTGAAgcaTTTAACGCtttCTTTACTT	-43	8.40	hypothetical protein

Table 10s. Predicted Pho boxes for PCC7942

Rank	Transcription unit	Names	PhoB boxes	Position	Score	Annotation of the first gene in the transcription unit
1	*Synpcc7942_1000*	*nucH*	TTTAAAGTgctGTTAATCCttcCTTTACCG	-310	9.72	hypothetical protein
2	*Synpcc7942_2445 Synpcc7942_2444 Synpcc7942_2443*	*sphX pstS pstC*	CTTAGGGTcgcCTTAATAGgctGTTAAACT	-46	9.71	hypothetical protein
	Synpcc7942_2442 Synpcc7942_2441	*pstA pstB*				
3	*Synpcc7942_0841*	-	GTTTAACCtgaCTTAATTCtgcATTGAATA	-75	9.45	putative flavoprotein involved in K+ transport
4	*Synpcc7942_1392*	*phoA*	TTTAACTAtttCATAATCTattCTCAATCT	-214	9.37	alkaline phosphatase
5	*Synpcc7942_2586*	-	GTTGATTTggcTTTAGTCTcctATTGATAT	-23	9.21	hypothetical protein
6	*Synpcc7942_1841 Synpcc7942_1840*	- -	ATTAACATagtCTTATCCTggcATTAGTCA	-88	9.15	UDP-N-acetylmuramoylalanyl-D-glutamyl-2,6-diaminopin
7	*Synpcc7942_0855 Synpcc7942_0856 Synpcc7942_0857*	- - -	TTTAACTTggcCTTAATCTtcgAATAGGTA	-87	9.04	response regulator receiver domain protein (CheY-like)
8	*Synpcc7942_0303*	-	TTTAAGCCcagCTTTAGCTcagCCTGGGAC	-20	8.91	response regulator receiver domain protein (CheY-like)
9	*Synpcc7942_2550*	-	TTTCTGCGgtcCTTAAACGagcCTTCTAGT	-36	8.90	hypothetical protein
10	*Synpcc7942_1649*	-	TTTGACAGcttTTTATCTAagaCTTATTCT	-34	8.77	rubrerythrin
11	*Synpcc7942_1253*	-	CTTAAACGgttCTTAACCTaagGGTTAACC	-141	8.71	hypothetical protein
12	*Synpcc7942_2486 Synpcc7942_2485*	- -	CTTCATTGagaCTTGACCTagcGCTTTGGT	-120	8.65	hypothetical protein
13	*Synpcc7942_2356*	-	ATAGAAAAggaATTAACCAaacCTTAAAGT	-84	8.60	putative transcriptional regulator, Crp/Fnr family
14	*Synpcc7942_1647*	-	GCTCAACAtctTTTCAATGattCCTGAATA	-96	8.47	hypothetical protein
15	*Synpcc7942_1957*	-	CTTAACCGcttCTGAATCTaagCCTGCTAT	-28	8.47	cyclic nucleotide-binding domain (cNMP-BD) protein
16	*Synpcc7942_1692*	-	GTTGACGGcaaGTTAAGTGaatTTTGCGAT	-43	8.46	mrr restriction system protein
17	*Synpcc7942_0169*	*glnN*	TCTAATTTtctCTTCAAAAggaATTTAAAT	-197	8.43	glutamate-ammonia ligase, glutamine synthetase type III
18	*Synpcc7942_0424*	-	CTTAGCTAaaaATTAAGGGtttTTTACACC	-81	8.32	photosystem q(b) protein
19	*Synpcc7942_0052 Synpcc7942_0051*	- -	GTTGGCACaaaATTCATCTtttACTAATCT	-125	8.30	hypothetical protein
20	*Synpcc7942_0557*	-	TTTCACTGagcCTTAACATtccCTTCGCGA	-126	8.21	hypothetical protein
21	*Synpcc7942_1565 Synpcc7942_1566*	- -	TTTTTGCCcatTTTAAATAactTTTGCTTT	-610	8.17	hypothetical protein
22	*Synpcc7942_2049 Synpcc7942_2048*	*psaA psaB*	CCTGAAAGctcTTTGAATTattGTTAATCG	-275	8.17	photosystem I core protein PsaA
23	*Synpcc7942_1134*	-	GTTGATGAgcaTTTCATTActcCTTCACTT	-378	8.13	hypothetical protein
24	*Synpcc7942_0489*	-	TTTAAGGAcaaGATATGCTggtTTTTACCA	-305	8.11	aldehyde dehydrogenase
25	*Synpcc7942_2139*	-	TTTATCATtttATTGAGCTtcaCTTACAGA	-513	8.03	probable glutathione S-transferase
26	*Synpcc7942_2152 Synpcc7942_2151*	- -	ATTTAAGAatgCTTGTTCTtaaAGTTGAAA	-19	8.02	hypothetical protein
27	*Synpcc7942_1357 Synpcc7942_1356 Synpcc7942_1355*	- - -	GTTAAACTcccCCTAAGCAcctTCTAGCTC	-43	7.99	multi-sensor signal transduction histidine kinase

Table 11s. Predicted Pho boxes for CC9605

Rank	Transcription unit	Names	PhoB boxes	Position	Score	Annotation of the first gene in the transcription unit
1	*Syncc9605_2251*	-	CTTAAGGCtgcGTTAATCTcgcTTTAACGT	-121	11.49	hypothetical protein
2	*Syncc9605_1831 Syncc9605_1832 Syncc9605_1833*	- - -	GCTGAACAtttCTTAAACCtggTTTACACT	-27	10.77	possible high light inducible protein
3	*Syncc9605_2479*	-	GTTGATATtttTTTAATGTcgcATTCGGCG	-248	9.89	conserved hypothetical protein
4	*Syncc9605_2586*	-	CTTTTCTTgatTTTCATCTaggTGTACTCT	-79	9.67	conserved hypothetical protein
5	*Syncc9605_2524*	-	TTCAACTCgtaATTAACTAgccCTTAAAGA	-370	9.43	VCBS
6	*Syncc9605_1478 Syncc9605_1477 Syncc9605_1476*	- - -	TTTCAACGcttCGTAAAGCtgtATGAAGCG	-53	9.33	conserved hypothetical protein
7	*Syncc9605_1396 Syncc9605_1395 Syncc9605_1394 Syncc9605_1393*	- - - -	CTTTTCTAgtcATTAAGAAcggCTTAAGCG	-56	9.26	molecular chaperone DnaK, heat shock protein HSP70
8	*Syncc9605_2373 Syncc9605_2372*	- -	CTAAACCTctgCTTAACCTgcgCTTCGCGC	-21	9.26	possible pectate lyase
9	*Syncc9605_2166 Syncc9605_2167*	- -	TTTAACCAcgcAATAATAAaacACTATAAA	-387	9.16	hypothetical protein
10	*Syncc9605_0307 Syncc9605_0306 Syncc9605_0305 Syncc9605_0304*	- - - -	GATGAAGGcttCTTAACAAcagCTTCATCA	-102	9.07	photosystem q(b) protein
11	*Syncc9605_0936 Syncc9605_0935*	- -	AGTAACAAgaaCTTACCCCtatTTTACGCT	-465	8.97	phage integrase family
12	*Syncc9605_0444 Syncc9605_0443 Syncc9605_0442*	- - -	AGTCGGACacaATTGATTGaggTTTATAAA	-60	8.96	phycobilisome linker polypeptide
13	*Syncc9605_0945*	-	CTTTATTAaatACTACCAGtgtTTTATCTA	-222	8.96	phage integrase family
14	*Syncc9605_2579 Syncc9605_2578*	- -	TTTATAAAttaCCTAATGTcgtATCAAATG	-130	8.92	possible phycobilisome rod-core linker polypeptide (L-RC 28.5)
15	*Syncc9605_1164 Syncc9605_1165 Syncc9605_1166 Syncc9605_1167*	- - - -	ATTCAAATaatATTATAATtggTTTAATTG	-63	8.91	hypothetical protein
	Syncc9605_1168 Syncc9605_1169 Syncc9605_1170	- - -				
16	*Syncc9605_0770*	-	GTTACCTTcaaATTGAGTTgcgATTGCATT	-4	8.84	hypothetical protein
17	*Syncc9605_1296 Syncc9605_1297 Syncc9605_1298 Syncc9605_1299*	- - - -	GTTTAAAActtCATCATCCtgcCGTAGATG	-45	8.81	hypothetical protein
18	*Syncc9605_1584*	-	ATTAAGTAtagCTTAAGATcgaATCAAAAG	-358	8.77	Flavodoxin, long chain
19	*Syncc9605_1580 Syncc9605_1581*	- -	ATTAAACAacaATTGAATGttgCCTGCCAA	-165	8.59	Ferredoxin (2Fe-2S)
20	*Syncc9605_1758 Syncc9605_1757*	- -	CTTAAGGAttgCTTAATTGtctCATGGTGA	-353	8.57	conserved hypothetical protein
21	*Syncc9605_2376 Syncc9605_2377*	- -	CATGAGAGgtaCTTCATGAtccGTTACATG	-70	8.54	hypothetical protein
22	*Syncc9605_0948*	-	CTTAATTTgttGATATCTTctgGTTAAATG	-558	8.53	hypothetical protein
23	*Syncc9605_1095*	-	CTTAACCGataCATAAACGaccCTTGGCTA	-101	8.50	possible porin
24	*Syncc9605_1413*	-	TTTTCTGCtagGTGAACACgacCTTCAGGC	-187	8.46	conserved hypothetical protein
25	*Syncc9605_1692 Syncc9605_1693 Syncc9605_1694 Syncc9605_1695*	- - - -	CTTTACCTgggCTTCAGAGcgcCTATCCCG	-86	8.45	hypothetical protein
26	*Syncc9605_1697 Syncc9605_1698*	- -	GTTAATGGgctCTTGCCCAgtgGTTCCTGC	-150	8.43	hypothetical protein
27	*Syncc9605_1274*	-	AGGAAAATgccCTTGATACaccCTTAAGTT	-292	8.41	conserved hypothetical protein
28	*Syncc9605_0148 Syncc9605_0147 Syncc9605_0146 Syncc9605_0145*	- - - -	ATTCAATGccaAATAATCTggcATTGAATG	-229	8.41	hypothetical protein
29	*Syncc9605_1756*	-	ATTTACAAaccGTTACAAGagcCTTAACAT	-23	8.40	possible high light inducible protein
30	*Syncc9605_1752*	-	CATAACCTtctCTTAATGAgacGGCAACTT	-39	8.39	possible pectate lyase
31	*Syncc9605_2395 Syncc9605_2394*	- -	GGTCAAGCcctTTTAAGGTtggTTTCACTT	-13	8.39	Carotene 7,8-desaturase
32	*Syncc9605_0815*	-	ATTCATCActtTTTTATTAagtATTCATTG	-159	8.38	Glycogen debranching enzyme GlgX
33	*Syncc9605_0459*	-	GTTCATCTgcgCATGAGCTgggTGTGATGA	-512	8.37	hypothetical protein
34	*Syncc9605_1397 Syncc9605_1398 Syncc9605_1399 Syncc9605_1400*	*pstC pstA pstB*	CTTAAGCCgttCTTAATGActaGAAAAGGG	-49	8.30	Phosphate ABC transporter, permease protein PstC

Table 12s. Predicted Pho boxes for MED4

Rank	Transcription unit	Names	Pho boxes	Position	Score	Annotation of the first gene in the transcription unit
1	*PMM0577 PMM0576 PMM0575*	- - -	CTTATTAAgaaATTAACTAtaaGTTAAATA	-21	9.28	Putative type II alternative sigma factor, sigma70 family
2	*PMM1708 PMM1709 PMM1710*	- *mraY* -	ATTAGAAAataTTTTAAATtttCTTTATCA	-61	9.10	hypothetical protein
3	*PMM0335*	-	CTTAAATTttaTTTAAATTtatTTTAATCT	-277	8.97	possible NADH-PLASTOQUINONE OXIDOREDUCTASE CHAIN 5 pir
4	*PMM0127*	-	CTTTCGATttgCTTAATAAttaCTTAATAA	-220	8.89	possible Signal peptide binding domain
5	*PMM0762 PMM0763 PMM0764 PMM0765*	*typA* - - -	AATAAGCAttcTTTGAGTTagcCTTAATAT	-28	8.83	tyrosine binding protein
6	*PMM0709*	*som*	ATTAATAGctaATTAATCTtctCTTAAACT	-241	8.58	possible porin
7	*PMM1167 PMM1166*	- -	TTTAAAATataTTTAGCATttaGTTAATTA	-251	8.21	Macrophage migration inhibitory factor family
8	*PMM0744*	-	ATTTCGTTgatATTTTTCTtaaCTTAATCT	-36	8.20	hypothetical protein
9	*PMM1495*	-	TTTCATATagaCTTAATTTtatATTTTAAA	-17	8.19	hypothetical protein
10	*PMM0999 PMM1000*	- -	ATTAGAAGataATTTATTAtttCTTAGCTA	-28	8.16	hypothetical protein
11	*PMM1355 PMM1356*	- -	TTTTACTTtaaCTTAATCTataGTAGAGAT	-63	8.15	hypothetical protein
12	*PMM0735*	-	TTTAAGTAattATTACAAGgacCTTAAATT	-17	8.14	hypothetical protein
13	*PMM1416*	-	TTTATAAAttaCTTAAATTtaaTTTAACCC	-74	8.11	hypothetical protein
14	*PMM0710*	*pstS*	TTTAACTAgccCTTAATCAtttCTTATATT	-245	8.07	ABC transporter, substrate binding protein, phosphate
15	*PMM0708 PMM0707*	*phoA* -	ATTTTTAAaacCTTAAATTgatCTTAATAA	-61	8.07	putative secreted protein
16	*PMM0348*	-	TTTGTAAGataTTTGATTTgtaCTTAATTC	-10	8.07	possible Spectrin repeat
17	*PMM1438 PMM1439*	*atpB atpC,atpE*	GTTAAGCAgcaATTAAACCactCTTTAACT	-105	7.85	ATP synthase beta subunit, central region:ATP synth...
18	*PMM0989*	-	CTTTGAATtaaTTTTACATatcCTTACTCT	-167	7.81	ATP/GTP-binding site motif A (P-loop)
19	*PMM1251 PMM1250*	- -	ATTGAAATgagTTTTATACtctCTTAAAAA	-145	7.78	Carbamoyltransferase

Table 13s. Predicted Pho boxes for MIT9312

Rank	Transcription unit	Names	Pho boxes	Position	Score	Annotation of the first gene in the transcription unit
1	*PMT9312_0577 PMT9312_0576 PMT9312_0575*	- - -	CTTATTACtgaATTAACTAtaaGTTAAATA	-20	9.29	putative type II alternative sigma factor, sigma70 family
2	*PMT9312_0289*	-	CTTTAAAAatgCCTGATATagaATTTATCC	-30	9.24	2-amino-4-hydroxy-6-hydroxymethyldihydropteridine pyrophosphokinase
3	*PMT9312_0721*	-	GTTAATTAgttCTTAACCTtctCTTAAAAC	-247	8.94	porin precursor-like
4	*PMT9312_0129*	-	CTTTTGATtttATTAATTAtttCTTAATAA	-198	8.91	signal peptide binding domain-like
5	*PMT9312_1453 PMT9312_1454*	- -	GTTCTTTAatcTTTTACTTtaaCTTAATCT	-62	8.78	conserved hypothetical protein
6	*PMT9312_1801 PMT9312_1802 PMT9312_1816*	- - -	ATAAAAAAgtaTTTTAAATttcCTTTATCA	-27	8.69	conserved hypothetical protein
7	*PMT9312_0709 PMT9312_0708*	- -	TTTAAACTataTTTAAACTtttCTTGATTC	-660	8.65	DnaJ central domain protein (4 repeats)-like
8	*PMT9312_0722*	*pstS*	TTTAAAGAgaaCTTAAACTtccCTTAAATT	-231	8.49	Periplasmic phosphate binding protein
9	*PMT9312_0356*	-	TTTTAGTTcttCTTAATTTagaTTTTTAGA	-185	8.18	spectrin repeat protein-like
10	*PMT9312_1179 PMT9312_1180*	- -	TTTTATAAtacTTTTATTAagcCATTAACT	-395	8.16	adhesin-like protein
11	*PMT9312_0787 PMT9312_0788*	- -	TTTTATTAaaaTTTAAACTaatCTTCTAGT	-18	8.06	Phosphoribosylformylglycinamidine synthetase PurS
12	*PMT9312_0340*	-	GTTTATTCtctCATATTCAgagCTTTTTTT	-330	8.05	NADH-plastoquinone oxidoreductase chain 5-like
13	*PMT9312_1588*	-	AATATTAAtatTTTTATATagaCTTAAGTT	-17	7.87	conserved hypothetical protein
14	*PMT9312_0819*	-	CTTAATTGctaATTAAACAataATTCAAGT	-334	7.87	S1 RNA binding domain-like
15	*PMT9312_0980*	-	CTTGAAGCagtATTTAAAAaatCTTAATTA	-69	7.86	conserved hypothetical protein
16	*PMT9312_1550 PMT9312_1549 PMT9312_1548 PMT9312_1547*	- - - -	TTTAATTGagaTTTTGCCAagtCTTTTATT	-208	7.85	ATP synthase subunit 1-like
	PMT9312_1546 PMT9312_1545 PMT9312_1544 PMT9312_1543	- - - -				
	PMT9312_1542 PMT9312_1541 PMT9312_1540	- - -				
17	*PMT9312_1007 PMT9312_1008*	- -	AATAAATGataATTAATTAtttATTAGCTA	-28	7.84	conserved hypothetical protein
18	*PMT9312_1135*	-	GTTTAAAAttaCTTAATTCttaTTTATAAA	-134	7.80	protein family PM-13
19	*PMT9312_0114 PMT9312_0115 PMT9312_0116*	- - -	TTTAATGTtttTTAAAACTtagCTAAAAAA	-33	7.75	conserved hypothetical protein
20	*PMT9312_1511 PMT9312_1510 PMT9312_1509 PMT9312_1508*	- - - -	ATTTAAGAataATTAAGTAgtcCTTTACTT	-329	7.70	hypothetical protein

Table 14s. Predicted Pho boxes for MIT9313

Rank	Transcription unit	Names	Pho boxes	Position	Score	Annotation of the first gene in the transcription unit
1	*PMT1985*	-	ATTAAGATtgtATTAATCTtccCTTAAGAC	-26	10.55	possible Pectate lyase
2	*PMT1986 PMT1987*	- -	CTTAAGGGaagATTAATACaatCTTAATTG	-552	9.28	conserved hypothetical protein in cyanobacteria
3	*PMT0624*	-	ATTCAACTtatCTTATTTAcaaATTTAGCA	-455	9.02	Uncharacterised protein family UPF0102
4	*PMT1046*	*pcb*	ATTATGGCaggGTTAATATtagCTTGATCT	-587	8.93	light-harvesting complex protein
5	*PMT1918 PMT1919*	- -	TTTGGGCTcacCTTAAATTcggTATTACCA	-15	8.91	hypothetical protein
6	*PMT0086*	-	TGTGAGACgtgATTTTTTTcgcGTTAAGCT	-23	8.88	Possible UDP-glucose-4-epimerase
7	*PMT1035*	-	ATTAATAGgttTTTAGGCTattTCTTAGGC	-315	8.60	hypothetical protein
8	*PMT0851*	-	GTTAACATttaCTTAAGATgcaTTTGCGCA	-751	8.55	hypothetical protein
9	*PMT0831 PMT0830*	- -	TTTTAGCCttcCGTAAAGGgcaCTTAGTGC	-386	8.50	hypothetical protein
10	*PMT0232*	*hupE*	TTTATTCAgcaATTAATTAgcaCTTAGCCA	-250	8.45	hypothetical protein
11	*PMT1023 PMT1024*	- -	CTAATTCTacgCTTAAGTAataCTTAAGCG	-316	8.37	hypothetical protein
12	*PMT1984*	-	TTTCTCCTggtCTTTATCCttcCTTAGGAT	-45	8.33	hypothetical protein
13	*PMT2212*	-	GTTAAATCtggCCTAGTCAaatCTTGACCT	-117	8.27	Class I peptide chain release factor
14	*PMT1098*	-	GTTGAGGTtcaCATTATGGgttTTTACTTG	-418	8.22	hypothetical protein
15	*PMT1435*	-	TTTGATAAaccATTTGTCGctcGTTAACCT	-160	8.22	hypothetical protein
16	*PMT0917*	-	ATTAAGGCtctGTTCAGCAataGGTTGGAG	-444	8.13	hypothetical protein
17	*PMT2126 PMT2127*	- -	TTTCACACcgtCTTCCGGTttcTTTGGTTT	-400	8.11	Phage integrase
18	*PMT1980*	-	CTTACTCTctgTTTAAACAcctCTTGGTGT	-284	8.10	hypothetical protein

Table 15s. Predicted Pho boxes for NATL2A

Rank	Transcription unit	Names	Pho boxes	Position	Score	Annotation of the first gene in the transcription unit
1	*PMN2A_0440*	-	CTTAACCAaacCATTACCTaacTTTAGTCA	-84	9.71	hypothetical protein
2	*PMN2A_0439 PMN2A_0438*	*phoA* -	CTTAACTTtttGCTAACCTtttTTTTAACT	-69	8.81	hypothetical protein
3	*PMN2A_0249*	-	CTTATTATttaTTTTATCTtttGTTAAACT	-64	8.61	hypothetical protein
4	*PMN2A_0435 PMN2A_0436 PMN2A_0437*	*Crp phoB phoR*	TATAATCAtatGTTAAACAgtaTTTAAGCT	-69	8.16	regulatory protein, Crp
5	*PMN2A_0549*	-	TTTGAGCCtaaTTTCACCCattCTTATCTC	-720	8.08	hypothetical protein
6	*PMN2A_0496*	-	TTTAAATActtCTTTCTCTctaCTTTACAG	-762	8.00	hypothetical protein
7	*PMN2A_0959*	-	TTTGAATAaagTTTGAATAactTTTGATCT	-151	7.80	enzyme of the cupin superfamily
8	*PMN2A_0559*	-	CTTAAAGCcctCTTTTTGAgggCTTTTTTT	-171	7.79	uncharacterized membrane protein
9	*PMN2A_0742*	-	ATTCCACCccaTTTAATTAcagGCTGATCC	-521	7.73	protein family PM-12
10	*PMN2A_1499*	-	CTTAAGCGaagGTTCACCAacaTTTAAAGA	-56	7.65	DNA repair protein RadA
11	*PMN2A_1369*	-	TTTAATGActaCTTTTGCAaatTGTGACAT	-287	7.54	hypothetical protein
12	*PMN2A_0714*	-	ATTAACAGgatCTTTCTATttaTTTAAGAT	-125	7.53	MIF/phenylpyruvate tautomerase family protein
13	*PMN2A_0311 PMN2A_0310 PMN2A_0309 PMN2A_0308 PMN2A_0307*	*pstC pstA pstB* - -	ATTAACCCtctCTTAGTGAaaaATTTGAAA	-47	7.52	hypothetical protein

Table 16s. Predicted Pho boxes for WH8102

Rank	Transcription unit	Names	Pho boxes	Position	Score	Annotation of the first gene in the transcription unit
1	*SYNW2391 SYNW2390*	*phoA1 phoA*	TTTGATCAgatCTTAAACTattCCTAACTT	-59	11.18	putative alkaline phosphatase
2	*SYNW2294 SYNW2293*	- -	CTTATAAAaaaATTAACTAtacATTAATTG	-131	10.22	Possible glycosyltransferase
3	*SYNW1664*	-	TTTATAGCaaaTTTAAGAGtcaCTTTATTA	-601	9.93	hypothetical protein
4	*SYNW0515 SYNW0516*	- -	GTTGAATTagtTTTAAGTTtgaTTTTGGAT	-121	9.74	hypothetical protein
5	*SYNW0443 SYNW0444 SYNW0445 SYNW0446 SYNW0447*	- - - - -	ATTTACGTttcTTTTTGCTttcTTTGTTGC	-234	9.67	possible oxidoreductase, GFO/Idh/MocA family protein
	SYNW0448 SYNW0449	*neuB neuC*				
6	*SYNW1344*	-	GTTGAACTcagCTTTATCCaatTTTACGCG	-113	9.49	hypothetical protein
7	*SYNW1144*	-	GTTTACGCtttCTTAACCTtgcTGTGGTGC	-516	9.40	phage integrase family
8	*SYNW2425 SYNW2426*	*gcvT aspS*	CTTGACCAaagCTTCACCCagaTTTAAGCA	-342	9.39	putative Glycine cleavage T-protein (aminomethyl transferase)
9	*SYNW0817 SYNW0816 SYNW0815*	*hli4 ubiH -*	AGGGAACAtttCTTAAACCtggTTTACACT	-25	9.13	possible high light inducible protein
10	*SYNW0305 SYNW0306*	*ftsH2 -*	CCTTAGGAcccGTTTAACAttcCTTCATCT	-16	9.09	hypothetical protein
11	*SYNW0085*	*swmA*	ATCAATATctcCTTAAATTtgaATTCACTT	-370	9.04	SwmA-cell surface protein required for swimming motility
12	*SYNW0363 SYNW0364 SYNW0365*	- - -	ATTAATGGaatGCTATTCTtagTTTATCGC	-24	9.02	hypothetical protein
13	*SYNW2423*	-	CTTAAGAGtttTTTATCTTgacTTTATTGA	-105	8.99	hypothetical, possible cyclic nucleotide-binding domain
14	*SYNW1019*	-	CTTGGACAaaaGTTAGACGggtCTTAACCG	-27	8.97	possible transcriptional regulator
15	*SYNW0886 SYNW0885 SYNW0884 SYNW0883*	- - - -	GTTGATGTggtGTTTATCCgccCTCAATGG	-105	8.95	hypothetical protein
16	*SYNW0894 SYNW0893 SYNW0892 SYNW0891*	- - - -	TATCAGCAtggATTCACCGagaGATGAGCG	-63	8.88	hypothetical protein
17	*SYNW0086 SYNW0087 SYNW0088*	*secA - -*	GTGAATTCaaaTTTAAGGAgatATTGATCG	-151	8.81	preprotein translocase SecA subunit
18	*SYNW1661*	-	GTTGGTTGgtcTTTCATCAgaaGATCAACC	-681	8.80	hypothetical protein
19	*SYNW1685*	-	GTTGAACCaatCCTAAGGAgggGTCACCAG	-29	8.80	putative SMR family multidrug efflux transporter
20	*SYNW0848 SYNW0849*	- -	ATTGCTAAtcaGTTAAGGAgtgCTTGTTGC	-594	8.76	hypothetical protein
21	*SYNW0246*	-	TTTCCCAGaatTTTCAAGTcccCTTAACCA	-15	8.71	two-component sensor histidine kinase
22	*SYNW2257 SYNW2256*	*crtD crtB*	GATCAGGCcctTTTAAGGTtggTTTCAACT	-13	8.60	hypothetical protein
23	*SYNW0642 SYNW0641*	- -	CTTTCCTAtcgATTGACTCggcATTAATAA	-79	8.59	hypothetical protein
24	*SYNW0385*	-	AGCCATCTgtaTTTAAATCagcGGTCACCT	-425	8.52	hypothetical protein
25	*SYNW0438*	-	ATTAAGATgtaTTTAACATctgATTTCAAA	-66	8.51	possible polysaccharide deacetylase (xylanase, chitin deacetylase)
26	*SYNW1999 SYNW2000 SYNW2001*	*cpeC - cpeE*	CTTGATTGaggTTTATAAAgaaCACGTTCT	-62	8.51	phycobilisome linker polypeptide
27	*SYNW0373 SYNW0372*	- -	ATTATACTcaaATTTACAGacaAATAAGTG	-4	8.51	hypothetical protein
28	*SYNW0984*	*expE1*	CTTAGGCCttcATTATCGAcctCTCCAATT	-55	8.51	putative secreted calcium-binding protein
29	*SYNW0360*	-	GTTTAGGCatcGTTGAACTaaaCTAAAGGA	-457	8.49	weak similarity to phage integrase family
30	*SYNW1533*	*gshR*	CTTCACGTgtcCTTGAGCCaatCTTGCTCA	-106	8.47	probable glutathione reductase (NADPH)
31	*SYNW2476 SYNW2475*	*- cobA*	TTTAAATCtgaTGTAAAACtctGTTCAGCA	-309	8.47	possible DNA mismatch repair proteins, mutS fa
32	*SYNW0450 SYNW0451 SYNW0452 SYNW0453 SYNW0454*	- - - - -	CTTAAATTcggCTTAAGTCaatTTAGTAAA	-236	8.46	putative sugar-phosphate nucleotide transferase
	SYNW0455 SYNW0456 SYNW0457 SYNW0458 SYNW0459	- - - - -				
	SYNW0460 SYNW0461	- -				
33	*SYNW2489*	-	CATCAAGTtgaTTTTAGACataTTTAAGAT	-603	8.45	hypothetical protein
34	*SYNW0165*	-	GTTAATAGatgCCTAATTTtttTTGGTCTG	-310	8.44	hypothetical protein
35	*SYNW0440 SYNW0441*	- -	GTTAAGCAcagATTGTGCAtatTTTCATGT	-303	8.43	hypothetical protein
36	*SYNW0389*	-	TTTGATCAgccCGTCATCTaaaTTTTATTG	-88	8.39	hypothetical protein
37	*SYNW2228*	-	CTTTACCAaggGTTAACCAataATCCCTGT	-109	8.37	hypothetical protein
38	*SYNW1563*	-	ATTGAGTTgttCCTGAACTagaATTTAGTT	-21	8.37	hypothetical protein
